# Whole-exome SNP array identifies 15 new susceptibility loci for psoriasis

**DOI:** 10.1038/ncomms7793

**Published:** 2015-04-09

**Authors:** Xianbo Zuo, Liangdan Sun, Xianyong Yin, Jinping Gao, Yujun Sheng, Jinhua Xu, Jianzhong Zhang, Chundi He, Ying Qiu, Guangdong Wen, Hongqing Tian, Xiaodong Zheng, Shengxiu Liu, Wenjun Wang, Weiran Li, Yuyan Cheng, Longdan Liu, Yan Chang, Zaixing Wang, Zenggang Li, Longnian Li, Jianping Wu, Ling Fang, Changbing Shen, Fusheng Zhou, Bo Liang, Gang Chen, Hui Li, Yong Cui, Aie Xu, Xueqin Yang, Fei Hao, Limin Xu, Xing Fan, Yuzhen Li, Rina Wu, Xiuli Wang, Xiaoming Liu, Min Zheng, Shunpeng Song, Bihua Ji, Hong Fang, Jianbin Yu, Yongxin Sun, Yan Hui, Furen Zhang, Rongya Yang, Sen Yang, Xuejun Zhang

**Affiliations:** 1 Institute of Dermatology and Department of Dermatology, No. 1 Hospital, Anhui Medical University, Hefei, Anhui 230022, China; 2 Department of Dermatology, Huashan Hospital, Fudan University, Shanghai 200040, China; 3 Department of Dermatology, No.2 Hospital, Anhui Medical University, Hefei, Anhui 230022, China; 4 Collaborative Innovation Center of Complex and Severe Skin Disease, Anhui Medical University, Hefei, Anhui 230032, China; 5 State Key Lab Incubation of Dermatology, Ministry of Science and Technology, Hefei, Anhui 230032, China; 6 Key Lab of Dermatology, Ministry of Education, Hefei, Anhui 230032, China; 7 Key Lab of Gene Resources Utilization for Severe Inherited Disorders, Anhui 230032, China; 8 Department of Dermatology, Peking University People’s Hospital, Beijing 100044, China; 9 Department of Dermatology, No.1 Hospital of China Medical University, Shenyang, Liaoning 110001, China; 10 Department of Dermatology, Jining No. 1 People’s Hospital, Jining, Shandong 272011, China; 11 Shandong Provincial Institute of Dermatology and Venereology, Jinan, Shandong 250022, China; 12 The Third People's Hospital of Hangzhou, Hangzhou, Zhejiang 310009, China; 13 Department of Dermatology, General Hospital of PLA Air Force, Beijing 100036, China; 14 Department of Dermatology, Southwest Hospital, Third Military Medical University, Chongqing 400038, China; 15 Department of Dermatology, Tianjin Changzheng Hospital, Tianjin 300106, China; 16 Department of Dermatology, Second Affiliated Hospital of Harbin Medical University, Harbin, Heilongjiang 150000, China; 17 Department of Dermatology, The Affiliated Hospital of Inner Mongolia Medical College, Huhehot, Inner Mongolia 010050, China; 18 Shanghai Skin Diseases and STD Hospital, Shanghai 200050, China; 19 Department of Dermatology, The First Affiliated Hospital of Dalian Medical University, Dalian, Liaoning 116011, China; 20 Department of Dermatology, The Second Affiliated Hospital, Zhejiang University School of Medicine, Zhenjiang 310009, China; 21 Department of Dermatology, Dalian Dermatosis Hosptial, Liaoning 116011, China; 22 Department of Dermatology, Yijishan Hospital of Wannan Medical College, Wuhu, Anhui 241000, China; 23 Department of Dermatology, The First Affiliated Hospital of Zhejiang University School of Medicine, Zhenjiang 310006, China; 24 Department of Dermatology, The First Affiliated Hospital of Zhengzhou University, Zhengzhou, Henan 450052, China; 25 Department of Dermatology, Anshan Tanggangzi hosptial, Liaoning 210300, China; 26 Department of Dermatology, First Affiliated Hospital of Xinjiang Medical University, Xinjiang 830054, China; 27 Department of Dermatology, General Hospital of Beijing Military Command, Beijing 100010, China

## Abstract

Genome-wide association studies (GWASs) have reproducibly associated ∼40 susceptibility loci with psoriasis. However, the missing heritability is evident and the contributions of coding variants have not yet been systematically evaluated. Here, we present a large-scale whole-exome array analysis for psoriasis consisting of 42,760 individuals. We discover 16 SNPs within 15 new genes/loci associated with psoriasis, including *C1orf141*, *ZNF683*, *TMC6*, *AIM2*, *IL1RL1*, *CASR*, *SON*, *ZFYVE16*, *MTHFR*, *CCDC129*, *ZNF143*, *AP5B1*, *SYNE2*, *IFNGR2* and 3q26.2-q27 (*P*<5.00 × 10^−08^). In addition, we also replicate four known susceptibility loci *TNIP1*, *NFKBIA*, *IL12B* and *LCE3D–LCE3E*. These susceptibility variants identified in the current study collectively account for 1.9% of the psoriasis heritability. The variant within *AIM2* is predicted to impact protein structure. Our findings increase the number of genetic risk factors for psoriasis and highlight new and plausible biological pathways in psoriasis.

## Abstract (web summary)

Psoriasis is a chronic inflammatory disease affecting up to 3% of the population. Here, Zuo *et al.* perform exome array analysis, identify single-nucleotide polymorphisms at 15 new loci, implicating several biological pathways in psoriasis risk and disease heritability.

## Graphical abstract

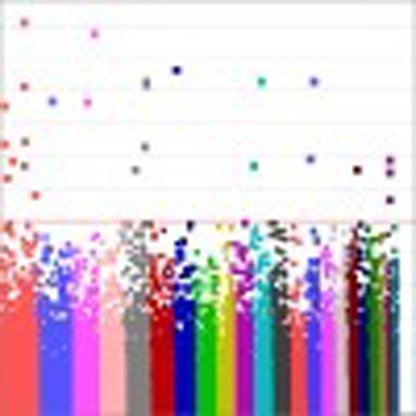

Psoriasis is a chronic inflammatory hyperproliferative cutaneous disease with dynamic interactions between the immune system and the epidermis that affects up to 3% of the population worldwide^1^,^2^,^3^. More than 40 susceptibility genes or loci have been identified for psoriasis in diverse populations, mostly through genome-wide association studies (GWASs)^4^,^5^,^6^,^7^,^8^,^9^,^10^,^11^,^12^,^13^,^14^,^15^,^16^,^17^. However, each of these identified genes or loci has a small or moderate effect, which only collectively explain a small proportion of the genetic variation in psoriasis. The ‘missing heritability’ in psoriasis is evident^18^. Furthermore, most of these previously identified variants are located in non-coding genomic regions^19^ and thus provide few clues as to the functional mechanism through which these variants affect the disease. Coding variants with high penetrance, which were poorly covered in conventional GWASs may contribute to finding the ‘missing heritability’ in complex disorders^20^,^21^,^22^.

Recent technological advances in high-throughput sequencing^23^ provide an opportunity to resequence multiple genetic regions and have generated compelling evidence that coding variants contribute to the mechanisms of psoriasis^5^,^24^ and other complex disorders^25^,^26^,^27^,^28^,^29^,^30^,^31^,^32^,^33^,^34^. However, these efforts to investigate coding variants are still limited due to sample size and thus the statistical power. Studies using new exome chips show their ability to comprehensively identify coding variants for several complex traits^34^,^35^,^36^.

Here, we perform the first exome-wide association study in large-scale individuals (17,614 cases and 25,216 controls) to systematically investigate the coding variants in psoriasis by using Illumina Human Exome Asian BeadChip (Exome_Asian Array) and Illumina Human Exome Fine Mapping BeadChip (Exome_Fine Array). A three-stage case–control design was implemented in the present study, and 15 new genes/loci were identified to associate with psoriasis. We also replicate four known susceptibility loci and all these 23 susceptibility variants identified in current study collectively account for 1.9% of the psoriasis heritability. These findings not only increase the number of genetic risk factors for psoriasis, but also highlight new and plausible biological pathways involved in this disease.

## Results

### Exome_Asian Array and Exome_Fine Array

In the first two stages, more than 260,000 markers were genotyped in two cohorts (Supplementary Tables 1 and 2), including 8,949 individuals (4,179 cases and 4,770 controls) and 13,473 individuals (7,066 cases and 6,407 controls), by using Exome_Asian Array and Exome_Fine Array, respectively. After quality control filtering and principal component analysis (PCA, online Methods; Supplementary Fig. 1), 108,576 and 108,099 variants were qualified in the Exome_Asian and Exome_Fine Arrays, respectively. In 11,245 cases and 11,177 controls, 87,827 non-MHC variants (77,641 nonsynonymous, nonsense or splice-site variants) were variable (Supplementary Fig. 2). A quantile–quantile plot and Manhattan plot were generated using Cochran–Armitage test for trend (Supplementary Figs 3 and 4). A clear deviation from the expected null distribution was observed in the quantile–quantile plot (Supplementary Fig. 3).

To validate the quality of our approach, we tested for association at known GWAS loci in Exome_Asian Array and Exome_Fine Array stages, respectively. Among the 88 previously described psoriasis-associated SNPs (*P*<5.00 × 10^−8^), 39 SNPs were directly genotyped on the Exome_Asian array with 24 SNPs pass the quality controls. Among these 24 SNPs examined, we identified 16 SNPs with significant or nominal association (*P*<0.05, Supplementary Table 3) through logistic regression (additive model). As for Exome_Fine Array stage, 38 SNPs were included and 23 of the SNPs pass the quality controls. Finally, 16 SNPs were replicated (*P*<0.05) in this stage (Supplementary Table 3). These results suggested that the genotype data from both of Exome_Asian Array and Exome_Fine Array were high reliability for our downstream analyses.

### Meta-analysis of the first two stages

We performed a meta-analysis of the first two stages on the 87,827 shared variants within the non-HLA region and identified five novel susceptibility loci through logistic regression (additive model), including *MTHFR* (rs2274976, *P*=2.33 × 10^−10^, odds ratio (OR)=0.79), *IL1RL1* (rs1420101, *P*=1.71 × 10^−10^, OR=0.88), *ZNF143* (rs10743108, *P*=1.70 × 10^−8^, OR=1.14), *ZNF683* (rs10794532, *P*=4.18 × 10^−8^, OR=1.11), and *TMC6* (rs12449858, *P*=2.28 × 10^−8^, OR=1.12) genes at genome-wide association significance and discovered four new variants within three known susceptibility loci, including *LCE3D–LCE3E* (rs10888501, *P*=6.48 × 10^−13^, OR=0.86), *IL12B* (rs1473247, *P*=5.63 × 10^−11^, OR=0.88; rs10076782, *P*=4.11 × 10^−11^, OR=0.88) and *NFKBIA* (rs12884468, *P*=1.05 × 10^−8^, OR=0.88) (Table 1 and Supplementary Figs 5 and 6). We performed conditional and linkage disequilibrium (LD) analyses to evaluate whether these significantly associated variants were independent of the established GWAS-identified SNPs in the Chinese population. Conditional analysis provided no evidence of association for these three loci containing secondary signals (Supplementary Table 4). For low-frequency variants (0.01< MAF <0.05), we did not identify any of them reaching genome-wide significance through single-variant analysis (Supplementary Data 1).

### Genotyping validation

To evaluate additional susceptibility genetic factors, we selected the top 76 SNPs with 5.00 × 10^−8^<*P*_*meta*_<1.00 × 10^−4^ (Table 2 and Supplementary Data 2) for further genotyping in an independent replication cohort of 6,369 cases and 13,969 controls (Supplementary Table 1). Meta-analysis of the 76 SNPs in the discovery (Exome_Asian Array stage and Exome_Fine Array stage) and replication stage studies identified 11 variants located in 11 diverse new susceptibility genes in the non-HLA region through logistic regression (additive model), including *NPPA* (rs5063, *P*=3.51 × 10^−9^, OR=0.85), *C1orf141* (rs72933970, *P*=1.23 × 10^−8^, OR=1.16), *AIM2* (rs2276405, *P*=3.22 × 10^−9^, OR=0.83), *CASR* (rs1042636, *P*=1.88 × 10^−10^, OR=0.91), *GPR160* (rs6444895, *P*=1.44 × 10^−12^, OR=1.11), *ZFYVE16* (rs249038, *P*=2.14 × 10^−8^, OR=0.84), *CCDC129* (rs4141001, *P*=1.84 × 10^−11^, OR=1.11), *AP5B1* (rs610037, *P*=4.29 × 10^−11^, OR=1.11), *SYNE2* (rs2781377, *P*=4.21 × 10^−11^, OR=0.85), *IFNGR2* (rs9808753, *P*=2.75 × 10^−8^, OR=0.92) and *SON* (rs3174808, *P*=1.15 × 10^−8^, OR=1.10; Table 2 and Supplementary Figs 5 and 6). Notably, two susceptibility coding variants were identified at 1p36 and located in *NPPA* (rs5063) and *MTHFR* (rs2274976), respectively. LD analysis revealed that these two variants are in a moderate LD (*D*′=0.70, *r*^2^=0.49) and further conditional analysis indicated that they are not independent signals from each other (rs5063, *P*_condition_=2.83 × 10^−1^, OR=0.94; rs2274976, *P*_condition_=2.60 × 10^−5^, OR=0.80; Supplementary Table 4). Similarly, we also identified two susceptibility coding variants at 21q22.11, which located in *IFNGR2* (rs9808753) and *SON* (rs3174808), respectively. Conditional and LD analyses showed that these two variants are in very mild LD (*D*′=0.76, *r*^2^=0.15) and independent from each other (rs9808753, *P*_condition_=1.22 × 10^−4^, OR=0.94; rs3174808, *P*_condition_=9.03 × 10^−4^, OR=1.06; Supplementary Table 4). In addition, we identified a missense variant (rs72933970) within *C1orf141*. At this region, multiple variants in or near *IL23R* have been identified to be associated with psoriasis. To reveal the relationship between rs72933970 and reported variants, we performed conditional and LD analyses and indicated that rs72933970 is an independent signal at this region (Supplementary Table 4). We also conformed three known genes, such as *LCE3D–LCE3E* (rs41268474, *P*=5.99 × 10^−11^, OR=1.17; rs76337351, *P*=1.71 × 10^−8^, OR=0.83), and *TNIP1* (rs10036748, *P*=4.26 × 10^−9^, OR=1.10; Table 2 and Supplementary Figs 5 and 6). Conditional and LD analyses were carried out to evaluate whether these SNPs were independent signals from the established GWAS-identified SNPs. Only one maker had limited impact on the associations at rs10036748 (*P*_*condition*_=2.04 × 10^−3^, OR=1.07) in (Supplementary Table 4). Furthermore, three suggestive loci 1q42.3, 10q22.3 and 21q22.11 were also identified with *P*_*meta*_<1.00 × 10^−06^.

## Discussion

In the present study, we identified 23 SNPs within 19 genes/loci associated with psoriasis, including 16 coding variants and 7 non-coding variants. Fifteen of 19 are newly identified genetic risk genes/loci, including *C1orf141*, *ZNF683*, *TMC6, AIM2*, *IL1RL1*, *CASR*, *SON*, *ZFYVE16*, *CCDC129*, *MTHFR*, *ZNF143*, *AP5B1*, *SYNE2*, *IFNGR2* and 3q26.2-q27, and remaining four are previously reported loci, such as *TNIP1*, *NFKBIA*, *IL12B* and *LCE3D–LCE3E*. These findings provide convincing evidence that common genetic variation is an important contributor to the risk of psoriasis.

All these newly identified loci are mini-effect ones, which needs large-scale samples to be detected. In this study, 17,614 cases and 25,216 controls are involved. Although 11 of 26 newly identified SNPs (Tables 1 and 2) are covered in Illumina Human610-Quad BeadChips used in our previous study^16^, because of the limited sample sizes (1,139 cases and 1,694 controls in our previous GWAS and the current study has much bigger sample size and thus power than previous studies), the *P* value are not significant enough (Supplementary Table 5). Therefore, these 11 SNPs were not chosen to validate in previous studies.

For 1p36, we identified two missense variants within *NPPA* (rs5063) and *MTHFR* (rs2274976). Condition analysis indicated that they represent the same signal and rs2274976 is more significant than rs5063 (Supplementary Table 4). *MTHFR* encodes a protein that acts as a co-substrate for homocysteinere methylation to methionine, which is important for maintaining the methyl donors for DNA methylation, thus resulting in gene regulation and cellular differentiation^37^.

For *AIM2* and *SYNE2*, we identified a stop-gained variant at each gene, including rs2276405 (*AIM2*) and rs2781377 (*SYNE2*). *AIM2* encodes a cytosolic double-stranded DNA (dsDNA) receptor. This receptor interacts with apoptosis speck-like protein to form a caspase-1-activating inflammasome and plays a putative role in tumorigenic reversion and controlling cell proliferation^38^. The protein encoded by *SYNE2* is a nuclear outer membrane protein that binds cytoplasmic F-actin and is a novel nesprin isoform that is expressed in skin^39^. A previous study demonstrated that the depletion of nesprin-2 reduces both the amount of active β-catenin inside the nucleus and T-cell factor/lymphoid-enhancing factor-dependent transcription^40^.

At 2q12.1, we identified a variant (rs1420101) within intron 3 of *IL1RL1.* The protein encoded by this gene is a member of the interleukin 1 receptor family, which have been proven to be involved in the function of helper T cells and this receptor can be induced by proinflammatory stimuli^41^. At 3q13, we discovered a missense variant at *CASR*. It encodes an endogenous calcium-sensing receptor (CaR), which is essential for mediating Ca(2+) signalling during Ca(2+)(o)-induced differentiation^42^. Extracellular Ca(2+) (Ca(2+)(o)) is a critical regulator that promotes differentiation in epidermal keratinocytes. The transforming growth factor beta signalling pathway is necessary for a variety of normal cellular processes^43^.

For *ZNF683*, *ZNF143* and *ZFYVE16*, we identified a missense variant on each gene, including rs10743108 (*ZNF143*), rs10794532 (*ZNF683*) and rs9808753 (*ZFYVE16*). It has been implicated that transcriptional regulatory proteins containing tandemly repeated zinc finger domains are thought to be involved in both normal and abnormal cellular proliferation and differentiation^44^,^45^. The *ZFYVE16* gene encodes an endosome protein that belongs to the FYVE zinc finger family of proteins. The encoded protein functions as a scaffold protein in the transforming growth factor beta signalling pathway and is involved in positive and negative feedback regulation of the bone morphogenetic protein signalling pathway^46^. In addition, we also discover a missense variant rs12449858 in *TMC6*, which encodes a member of the EVER protein family, which may be involved in the regulation of cellular zinc homeostasis in lymphocytes and which have been considered as key components of the activation-dependent regulation of Zn(2+) concentration in T cells^47^.

At 21q22.11, we identified two susceptibility coding variants, which were located in *IFNGR2* (rs9808753) and *SON* (rs3174808), respectively. Conditional and LD analyses showed that these two variants are independent from each other. *IFNGR2* encodes the non-ligand-binding beta chain of the gamma interferon receptor, which may affect the generation of Th17 cells from memory T cells^48^,^49^. However, the biological function of *SON* is unclear. In addition, four new susceptibility genes (*C1orf141, GPR160*, *CCDC129* and *AP5B1*) with unknown functions in the pathogenesis of psoriasis were also identified, indicating that additional molecular mechanisms contribute to the risk of developing psoriasis. Therefore, further studies are required to fully understand how variations in these genes are involved in the pathogenesis of psoriasis.

The restricted maximum-likelihood method^18^ indicated that the susceptibility variants identified in this study together explained 1.9% of the variance in psoriasis heritability. Fifteen of them are nonsynonymous variants and seven were predicted to be damaging by either SIFT or PolyPhen software (Supplementary Table 6). In addition, amino acid residue 32 of *AIM2* (rs2276405) was found to be located in the middle of an alpha-helix motif buried inside the protein structure (Supplementary Fig. 7). The wild-type Glu residue is acidic, but the mutant Lys residue is basic. As the chemical properties of Glu and Lys are completely opposite, this substitution may destabilize the alpha-helix motif. Five of these fifteen newly identified non-HLA genes were shown to be significantly enriched in the network (*MTHFR*, *NPPA*, *AIM2*, *CASR* and *IFNGR2*; FDR <0.1) (Supplementary Fig. 8).Gene expression analysis on the basis of public psoriasis databases^38^ indicated that most of the susceptibility genes, newly identified or confirmed in this study, display highly differential expression in skin from psoriatic patients compared with normal controls (Supplementary Table 7). We also performed functional annotations on the basis of the ENCODE data set for these 26 newly identified SNPs (Supplementary Data 3), and showed that most of these SNPs fall within promoters, enhancers, Dnase hypersensitive sites and transcription factor binding sites.

This study was designed to maximize statistical power in a cost-effective manner by adopting a multi-stage analysis strategy for a large-scale Han Chinese population resulting in the identification of 15 new susceptibility genes/loci for psoriasis. Our findings highlight the genetic contributions of common variants to the pathogenesis of psoriasis and increase the number of known genetic risk factors for psoriasis. This study also highlights new and plausible biological pathways in psoriasis, thereby suggesting additional genetic factors that may contribute to the genetic heterogeneity of psoriasis in the Han Chinese population. Further study will be needed to understand the molecular mechanisms underlying these risk variants identified in this study in the aetiology of psoriasis.

## Methods

### Study design and study samples

We implemented a three-stage case–control design in this study (Supplementary Fig. 2). The subjects, consisting of 17,614 psoriatic cases and 25,146 healthy controls, were enrolled through a collaborative consortium in China (Supplementary Table 1). All the cases were diagnosed by at least two dermatologists, and their clinical information was collected through a comprehensive clinical check-up by professional investigators. In addition, demographic information was collected from all the participants through a previously described structured questionnaire^16^. All the healthy controls were clinically determined to be without psoriasis, any autoimmune disorders and systemic disorders or any family history of psoriasis and other autoimmune-related disorders (including first-, second- and third-degree relatives). Cases and controls were well matched for both age and sex. All samples were self-reported Han Chinese. Written, informed consent was given by all the participants. The study was approved by the institutional ethics committee of each hospital (The Second Hospital of Anhui Medical University, The First Affiliated Hospital of Anhui Medical University and Huashan Hospital of Fudan University) and was conducted according to the Declaration of Helsinki principles.

### Exome array and genotyping in first two stages

In this study, we used two exome array types as follows: custom Illumina Human Exome Asian BeadChip (Exome_Asian Array) and Illumina Human Exome Fine Mapping BeadChip (Exome_Fine Array). The former platform includes 242,102 markers focused on putative functional coding variants from >12,000 exome and genome sequences representing multiple ethnicities and complex traits in addition to >30,000 Chinese population-specific coding variants, identified by whole-exome sequencing performed in 781 psoriasis cases and 676 controls by our group^5^. The latter includes all markers in the Illumina Human Exome-12v1_A BeadChip and 28,139 coding variants in 185 susceptibility genes, which have been reported in immune-related disease GWASs. The details of the SNP content and selection strategies are described on the exome array design webpage ( http://genome.sph.umich.edu/wiki/Exome_Chip_Design).

In this study, two cohorts, including 8,949 samples (4,179 cases and 4,770 controls) and 13,473 samples (7,066 cases and 6,407 controls), in addition to 100 blind duplicate samples, were genotyped using the Exome_Asian Array and Exome_Fine Array, respectively. The genotyping was conducted at the State Key Lab Incubation Base of Dermatology, Ministry of National Science and Technology (Anhui Medical University). The genotype calling and the clustering of study sample genotypes were performed using Illumina’s GenTrain (version 1.0) clustering algorithm in Genome Studio (version 2011.1).

### Quality controls

We excluded 204 samples (90 cases and 114 controls) with genotyping call rates <98% in individuals during the first two stages. We then examined potential genetic relatedness on the basis of pairwise identity by state for all the successfully genotyped samples using PLINK 1.07 software^50^. On the identification of a first- or second-degree relative pair, we removed one of the two related individuals (the sample with the lower call rate was removed). We defined close relatives as those for whom the estimated genome-wide identity-by-descent proportion of alleles shared was >0.10. In total, 87 samples (33 cases and 54 controls) were removed due to sample duplication and genetic relatedness. The remaining samples were subsequently assessed for population outliers and stratification using a PCA-based approach^51^. For all PCA, all HLA SNPs on chr.6: 25–34 Mb and SNPs on non-autosomes were removed (Supplementary Fig. 1). Furthermore, we excluded SNPs with a call rate <99%, a minor allele frequency (MAF) <0.0001 and/or a significant deviation from Hardy–Weinberg equilibrium (HWE) in the controls (*P*<10^−4^) during each stage. We computed principal components of Exome_Asian Array (including 13,473 individuals) and Exome_Fine Array (including 8,949 individuals) stages using 108,576 SNPs and 108,099 SNPs (MAF >0.0001, SNPs with HWE *P*>10^−4^, SNPs with a call rate >99%, and carrying out LD pruning using the PLINK option ‘-indep-pairwise 50 5 0.2′), respectively. After quality control, the genotype data of 89,720 overlapped autosomal variants in 11,245 cases and 11,177 controls were included for further analysis.

### Genotyping comparison

To evaluate the genotyping quality, we compared the concordance rates for the samples genotyped in our study and either (i) samples sequenced by whole-exome sequencing in our previous study^1^ or (ii) samples genotyped on the Illumina Human610-Quad BeadChip^16^. For the Exome_Fine Array data, the comparisons were based on 89,720 and 12,320 overlapping variants within 102 and 38 individuals, respectively. The concordance rates were 99.985 and 99.978% for the whole-exome sequencing data and Illumina Human 610-Quad BeadChip data, respectively. Moreover, the concordance rates for the homozygous and heterozygous genotypes were 99.975 and 99.965%, respectively, for the whole-exome sequencing data and 99.874 and 99.954%, respectively, for the Illumina Human 610-Quad BeadChip data. For the Exome_Fine Array data, the comparisons were based on 15,620 and 22,458 overlapping variants within 348 and 159 individuals, respectively. The concordance rates were 99.964 and 99.986% for the whole-exome sequencing data and the Illumina Human610-Quad BeadChip data, respectively. Moreover, the concordance rates for homozygous and heterozygous genotypes were 99.978 and 99.968%, respectively, for the whole-exome sequencing data and 99.865 and 99.976%, respectively, for the Illumina Human 610-Quad BeadChip data. The concordance rate of the 100 blind duplicate samples was 99.988%.

### SNP selection and genotyping for replication

To replicate the association results of the meta-analysis of the Asia array and the Fine Mapping array, we further analysed the 76 top variants in an additional 20,338 samples (6,369 cases and 13,969 controls, Supplementary Table 1) using the Sequenom MassARRAY system. All of these selected SNPs met the following quality criteria: (1) the MAF was higher than 0.5% in both the cases and controls; (2) HWE in the controls was *P*≥0.01 and the HWE in the cases was *P*>10^−4^; (3) SNPs with a meta-association of *P*<10^−4^ after adjustment for gender; (4) proximity to putative candidate genes (immune-related or involved in immune cell proliferation and differentiation) or known susceptibility loci for autoimmune diseases; and (5) in each locus, one or two of the most significant SNPs were selected for validation. For all of the 76 SNPs analysed in the validation study, the cluster patterns of the genotyping data from the Illumina and Sequenom analyses were checked to confirm their high quality. The genotype data are available in Table 2 and Supplementary Data 2.

### Statistical analyses

*Single-variant analysis*. Single-marker association analyses were performed to test for disease–SNP associations, assuming an additive allelic effect and using logistic regression in each stage. The Cochran–Armitage trend test was conducted in these two-stage samples. We performed heterogeneity tests (*I*^*2*^ and *P* values of the *Q* statistics) between the two groups using the Breslow–Day test^52^, and the extent of heterogeneity was assessed using the *I*^*2*^ index^53^. To improve the statistical power, we combined the association results in the first two stages using meta-analysis. The fixed effect model (Mantel–Haenszel) was applied when *I*^*2*^ was <30% (ref. 54). Otherwise, the random effect model (DerSimonian–Laird) was implemented^55^.

*Conditional analysis*. We carried out conditional analyses to identify additional association signals after accounting for the effects of known and newly discovered susceptibility loci. To investigate more than two association signals per locus, we used a stepwise procedure in which additional SNPs were added to the model according to their conditional *P* value, as programmed in EMMAX^56^. We estimated the LD metrics *r*^2^ and *D*’ using 9,633 individuals from METSIM who passed genotyping quality control. LD with SNPs not included on the exome array was determined on the basis of exome sequence or targeted sequence data for 21,309Han Chinese individuals.

*Annotation*. Annovar^57^ was used to functionally annotate the SNPs according to their location and their expected effect on encoded gene products on the basis of information from the RefSeq database.

### Protein structure analysis

We searched for published three-dimensional protein structures in the Research Collaboratory for Structural Bioinformatics Protein Data Bank (RCSB PDB; http://www.rcsb.org/pdb/home/home.do) and downloaded structure for *AIM2* (3VD8). We used DeepView/Swiss-Pdb Viewer ( http://www.expasy.org/spdbv/) to view the protein structures and to examine the side chains of the original and mutant residues at the relevant amino acids. SIFT^58^ was used to predict the damage evolution and progression for associated nonsynonymous variants.

### Statistical analysis of networks

To identify the proximal interactors, we expanded the global network by including the Human Net protein interaction database^59^ and literature-curated interactions from STRING^60^,^61^ to derive an expanded global network based on known protein–protein interactions using the previously published candidate gene-based and GWAS-based data.

## Additional information

**Accession codes:** The variant data for psoriasis cases and controls mentioned in this study have been deposited in dbVar under the accession code SUB822238.

**How to cite this article:** Zuo, X. *et al.* Whole-exome SNP array identifies 15 new susceptibility loci for psoriasis. *Nat. Commun.* 6:6793 doi: 10.1038/ncomms7793 (2015).

## Supplementary Material

Supplementary Information Supplementary Figures 1-8 and Supplementary Tables 1-7

Supplementary Data 1 The results of the low-frequency (0.01< MAF <0.05) using single variant analysis.

Supplementary Data 2 Meta-analysis of the 59 SNPs in the discovery (Exome_Asian Array stage and Exome_Fine Array stage) and replication stage.

Supplementary Data 3 The functional annotations based on the ENCODE dataset for 26 newly identified SNPs.

## Figures and Tables

**Table 1:**
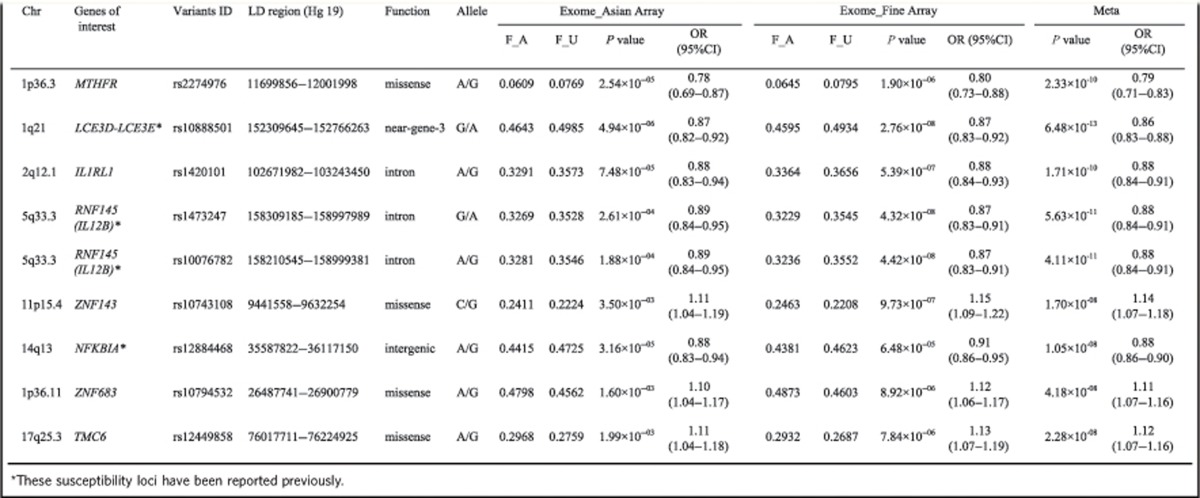
Association results from the first two stages (Exome_Asian Array and Exome_Fine Array) through logistic regression (additive model).

**Table 2:**
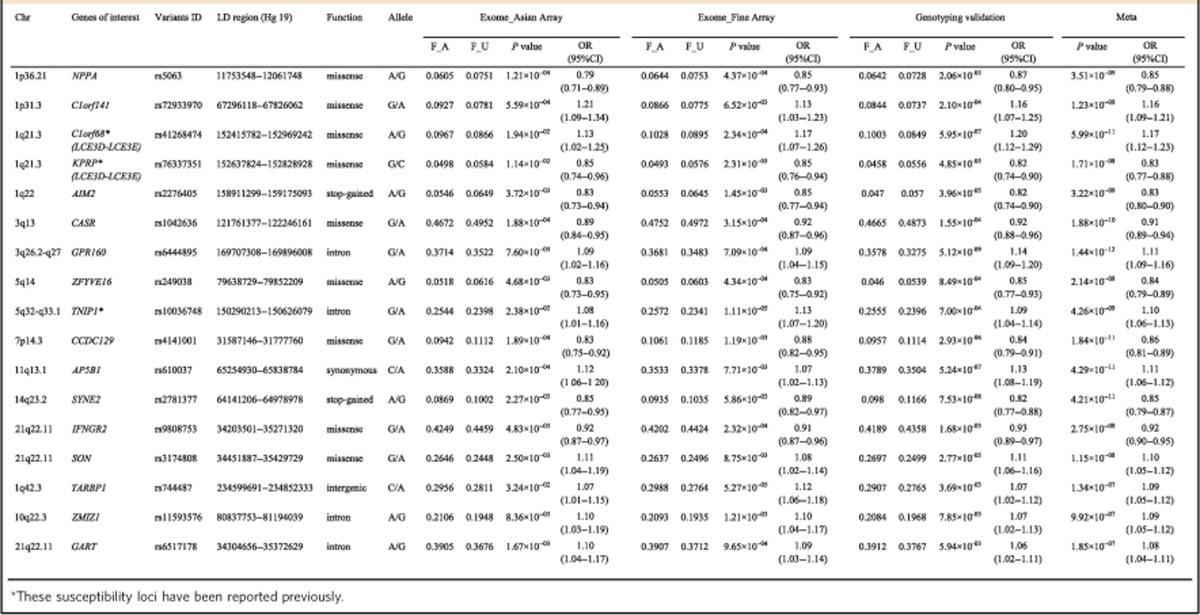
Association results from each of the three stages and combined analyses through logistic regression (additive model).
