# Author Correction: Whole-exome SNP array identifies 15 new susceptibility loci for psoriasis

**DOI:** 10.1038/ncomms16186

**Published:** 2018-03-13

**Authors:** Xianbo Zuo, Liangdan Sun, Xianyong Yin, Jinping Gao, Yujun Sheng, Jinhua Xu, Jianzhong Zhang, Chundi He, Ying Qiu, Guangdong Wen, Hongqing Tian, Xiaodong Zheng, Shengxiu Liu, Wenjun Wang, Weiran Li, Yuyan Cheng, Longdan Liu, Yan Chang, Zaixing Wang, Zenggang Li, Longnian Li, Jianping Wu, Ling Fang, Changbing Shen, Fusheng Zhou, Bo Liang, Gang Chen, Hui Li, Yong Cui, Aie Xu, Xueqin Yang, Fei Hao, Limin Xu, Xing Fan, Yuzhen Li, Rina Wu, Xiuli Wang, Xiaoming Liu, Min Zheng, Shunpeng Song, Bihua Ji, Hong Fang, Jianbin Yu, Yongxin Sun, Yan Hui, Furen Zhang, Rongya Yang, Sen Yang, Xuejun Zhang

Nature Communications 6: Article number: 6793; DOI: 10.1038/ncomms7793 (2015); Published: 04 09 2015; Updated: 03 13 2018

In the originally published version of this Article, there were errors in the Methods section and in Supplementary Figure 2. In the Methods section entitled ‘Quality control’, and in Supplementary Figure 2, references to ‘MAF>0.0001’ were incorrectly given as ‘MAF>0.01’. These errors have now been corrected in both the PDF and HTML versions of the Article and in the Supplementary Information File.

