# Supplementary material for: Whole-exome SNP array identifies 15 new susceptibility loci for psoriasis
Source: Nat Commun. 2015 Apr 9;6:6793. doi: 10.1038/ncomms7793 (PMC4403312; doi:10.1038/ncomms7793)
Supplement: Supplementary Information — Supplementary Figures 1-8 and Supplementary Tables 1-7 [file ncomms7793-s1.pdf]

Supplementary Figures:

(a): Exome_Asian Array.

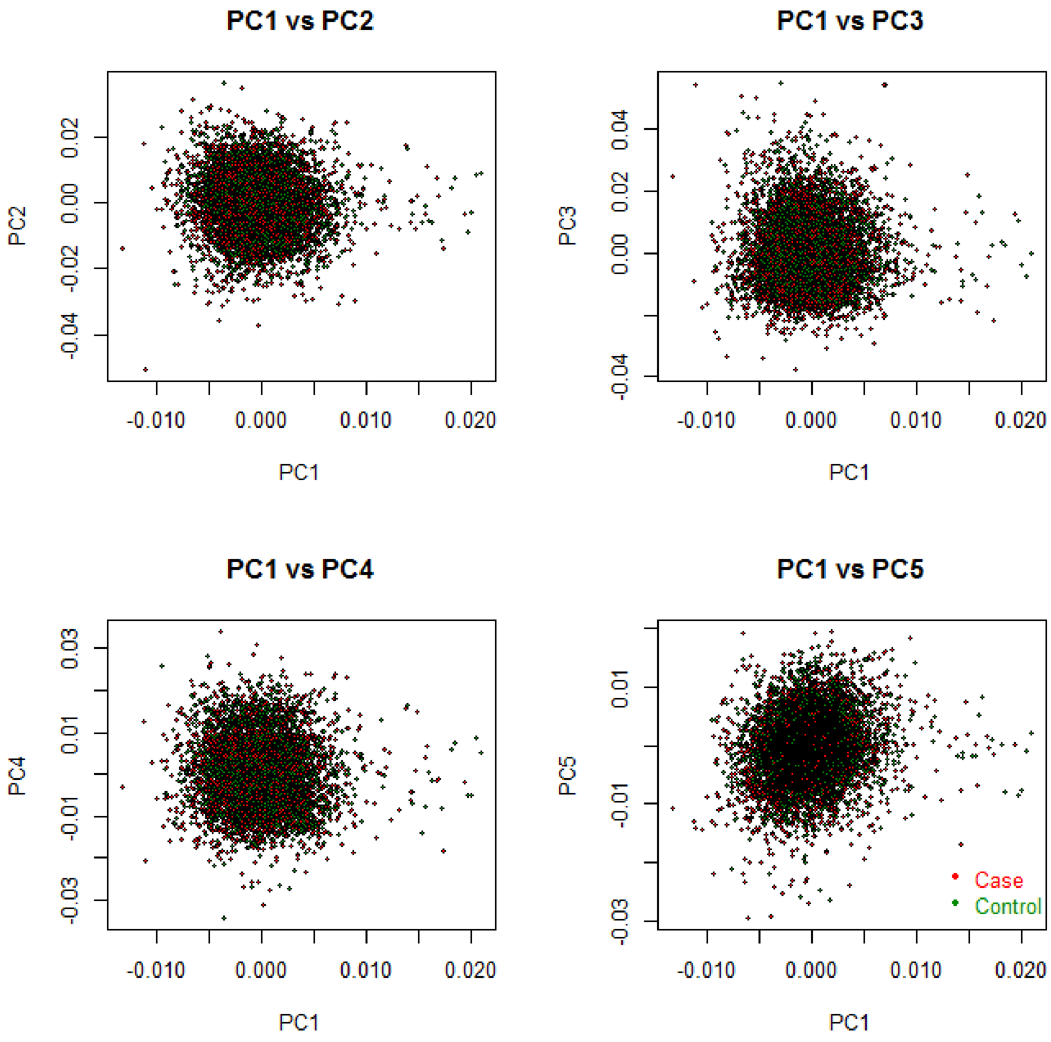

(b): Exome_Fine Array

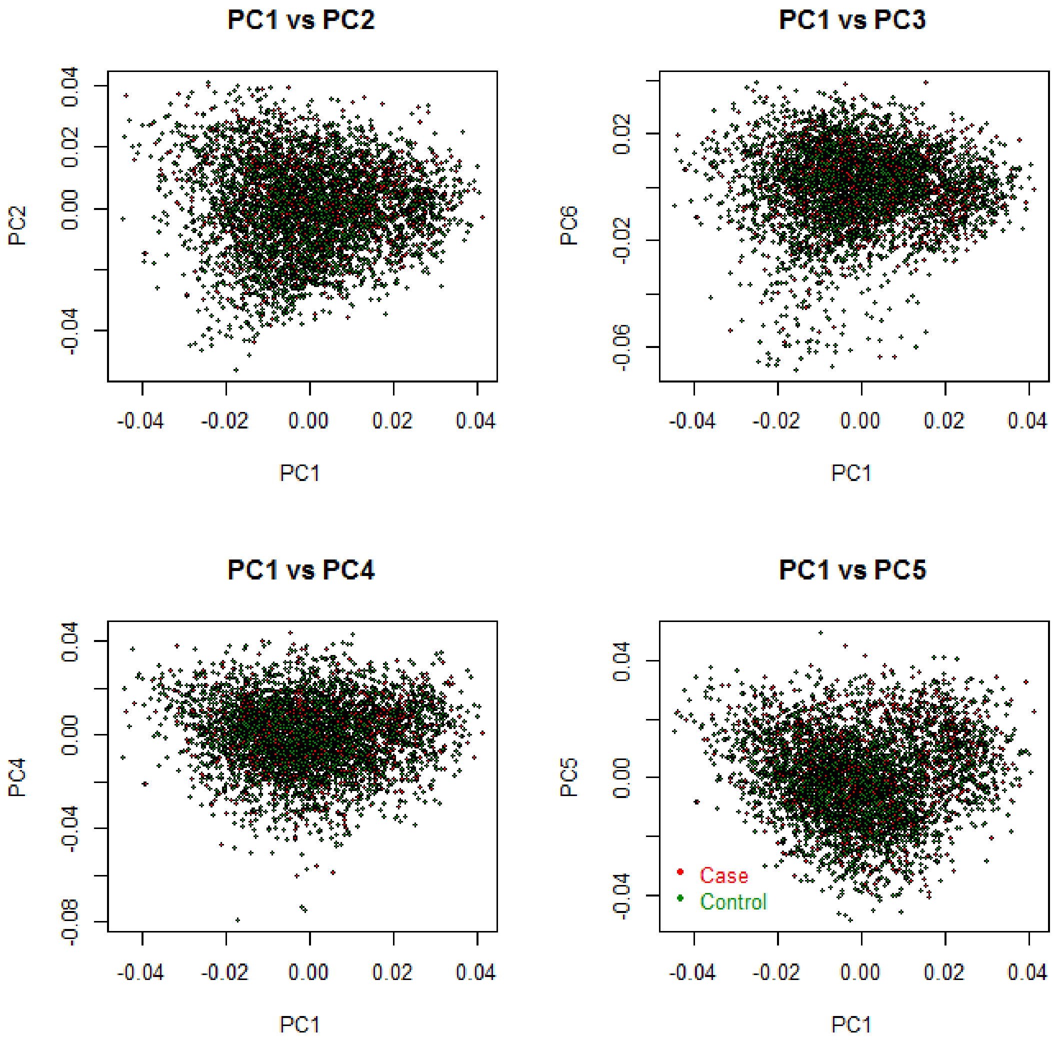

Supplementary Figure 1| The principal components analysis (PCA) were performed in 8,949 samples (4,179 cases, 4,770 controls) in Exome_Asian Array stage and 13,473 samples (7,066 cases, 6,407 controls) in Exome_Fine Array stage, respectively. (a): Exome_Asian Array, (b): Exome_Fine Array.

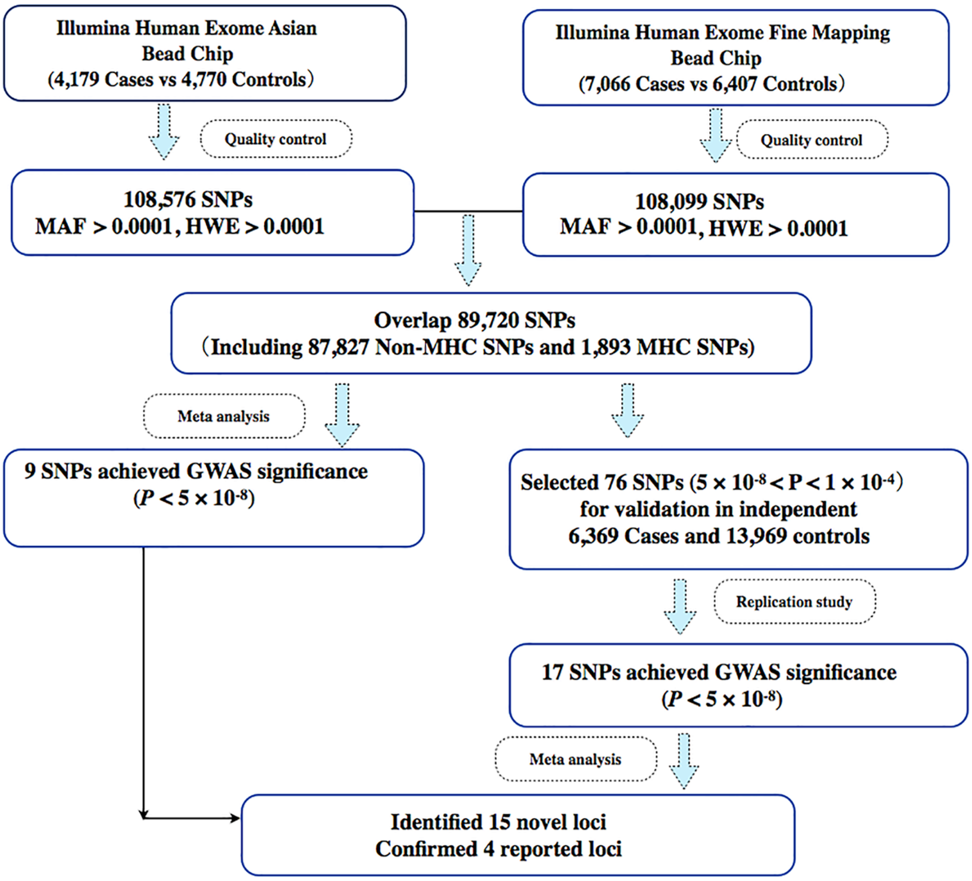

Supplementary Figure 2 | Study design: In the first two stages, more than 270,000 markers were genotyped in two cohorts, including 8,949 individuals (4,179 cases and 4,770 controls) and 13,473 individuals (7,066 cases and 6,407 controls), by using Exome_Asian Array and Exome_Fine Array, respectively. After quality control filtering and principal component analysis (Online Methods), 108,576 and 108,099 variants were qualified in the Exome_Asian and Exome_Fine Arrays, respectively. In 11,245 cases and 11,177 controls, 87,827 non-MHC variants were variable. We performed a meta-analysis of the first two stages on the 87,827 shared variants within the non-HLA region and identified 9 SNPs reached genome-wide significance level (P $< 5.00 \times 10^{-08}$). In order to evaluate additional susceptibility genetic factors, we

selected the top 76 SNPs with $5.00 \times 10^{-8} < P_{meta} < 1.00 \times 10^{-4}$ for further genotyping in an independent replication cohort of 6,369 cases and 13,969 controls through the Sequenom MassARRAY system. Meta-analysis of these 76 SNPs in the discovery and replication stage studies identified 17 variants achieved genome-wide significance level.

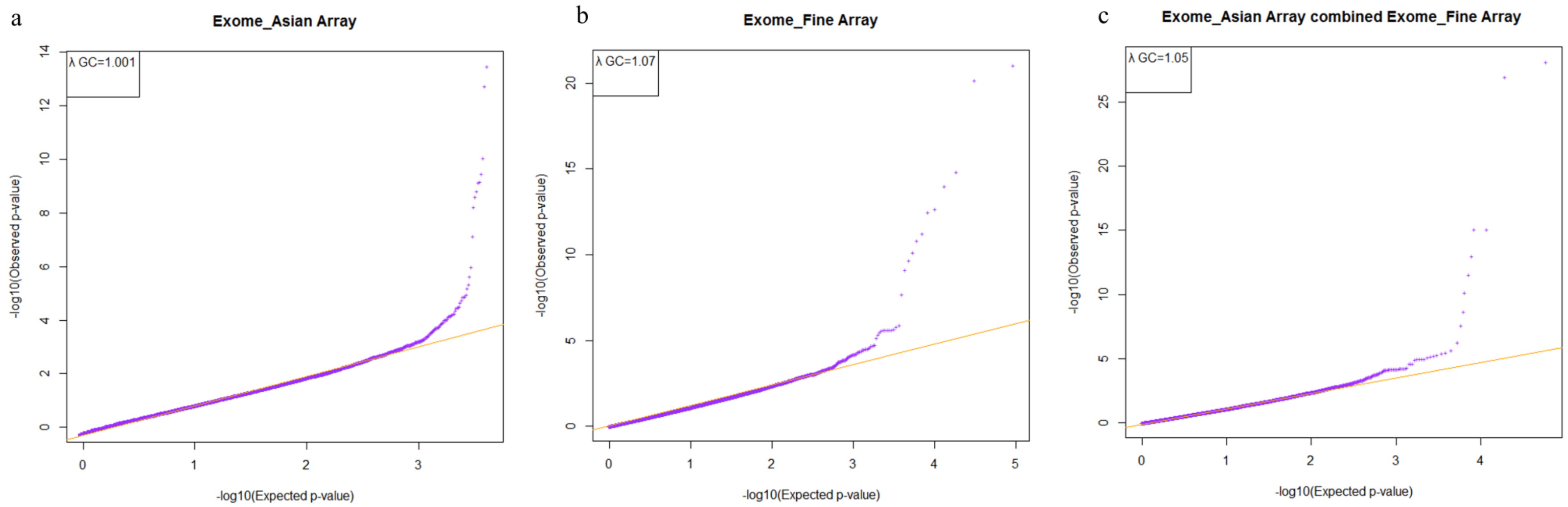

Supplementary Figure 3 | Quantile-quantile plots of the observed *P* values versus the expected null distribution of *P* value (after adjust PC1-5) of association in the first two stages, including 8,949 individuals (4,179 cases and 4,770 controls) in the first stage and 13,473 individuals (7,066 cases and 6,407 controls) in the second stage. **a.** The first stage using Exome_Asian Array. The plot was obtained after removing 2,329 SNPs within MHC region (Chr. 6: 25–37 Mb). **b.** The second stage using Exome_Fine Array. The plot was obtained after removing 2,760 SNPs within MHC region (Chr. 6: 25–37 Mb). **c.** The meta analysis. The plot was obtained 87,827 SNPs.

(a)

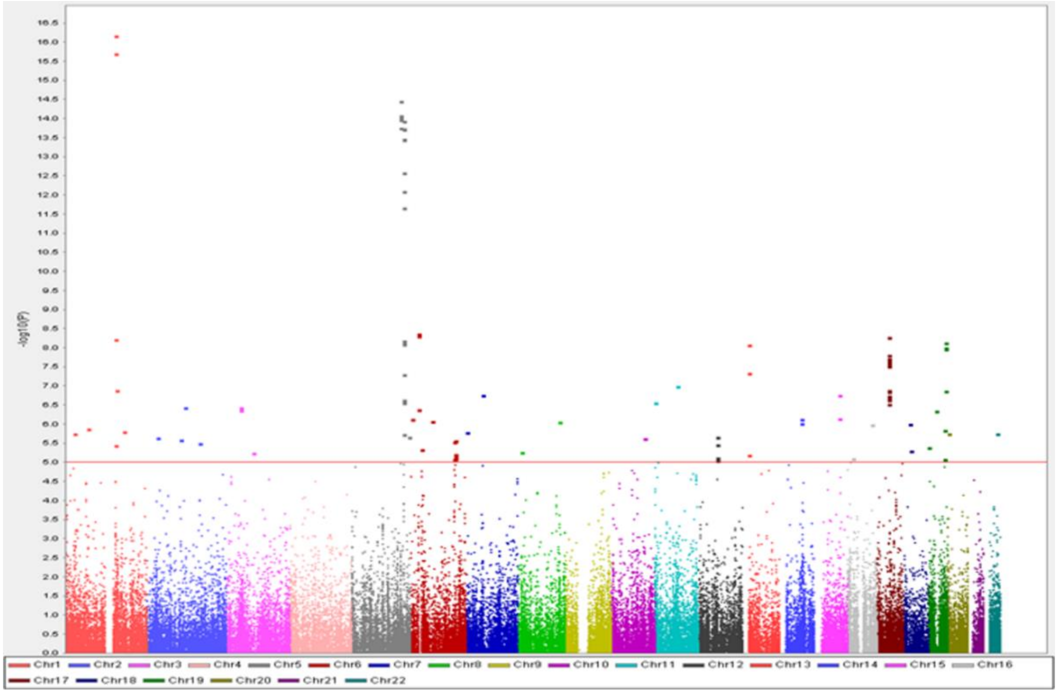
-log10(P)
Chr1 Chr2 Chr3 Chr4 Chr5 Chr6 Chr7 Chr8 Chr9 Chr10 Chr11 Chr12 Chr13 Chr14 Chr15 Chr16
Chr17 Chr18 Chr19 Chr20 Chr21 Chr22

(b)

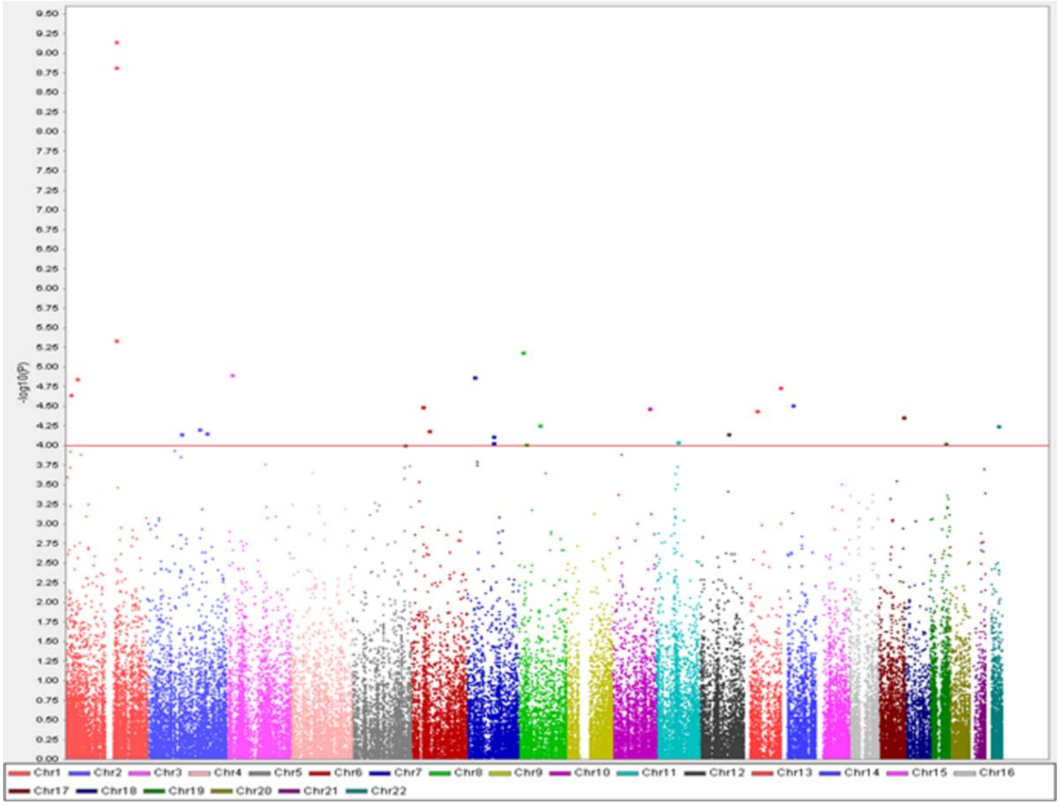
-log10(P)
Chr1 Chr2 Chr3 Chr4 Chr5 Chr6 Chr7 Chr8 Chr9 Chr10 Chr11 Chr12 Chr13 Chr14 Chr15 Chr16
Chr17 Chr18 Chr19 Chr20 Chr21 Chr22

(c)

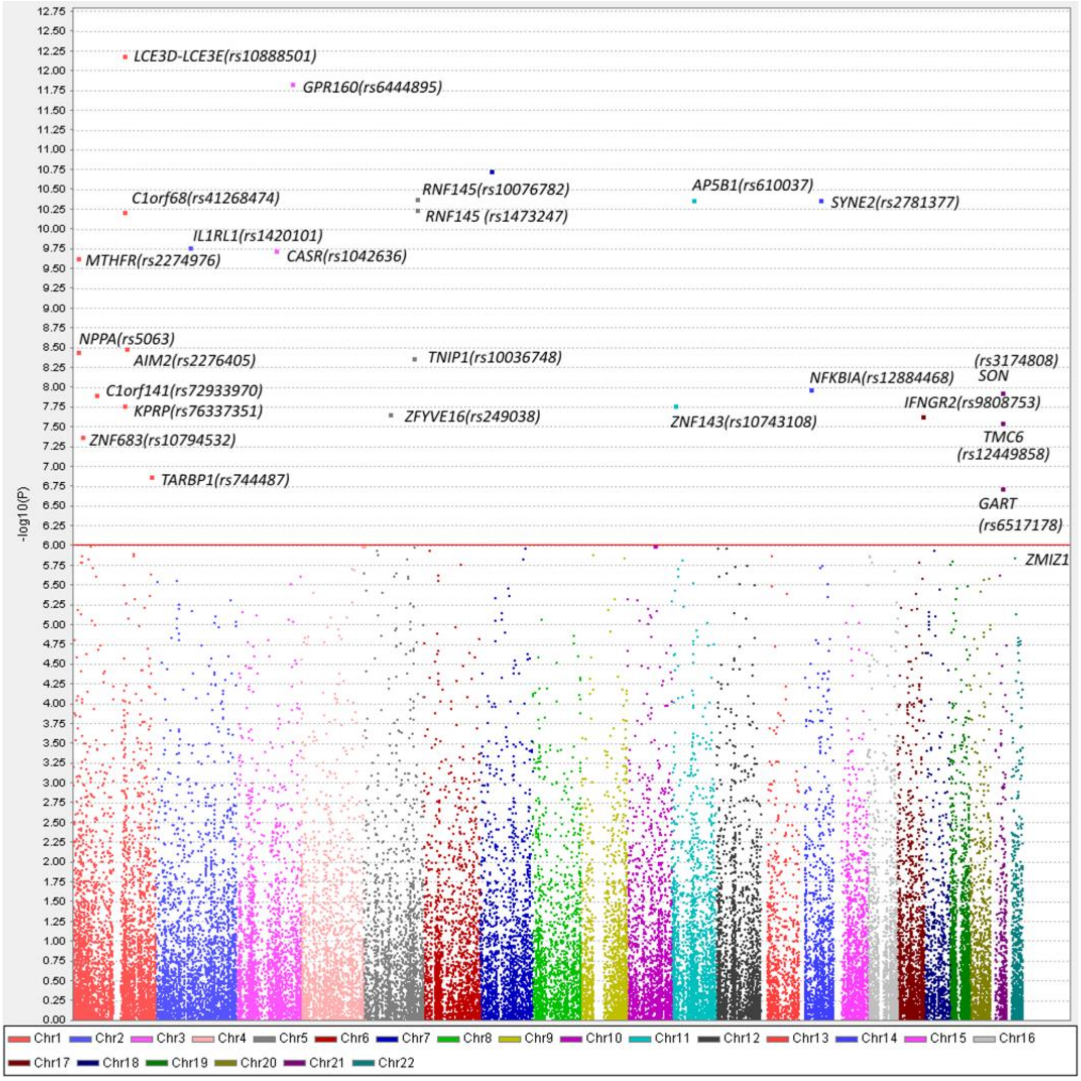

Supplementary Figure 4 | Manhattan plot of the association evidences in the first two stages. (a). 108,099 SNPs in the first stage (4,179 cases and 4,770 controls) using Exome_Fine Array. (b). 108,576 SNPs in the second stage (7,066 cases and 6,407 controls) using Exome_Asian Array. (c). 89,720 SNPs in the meta analysis, including 11,245 cases and 11,177 controls.

(a): 1p36.3 (*MTHFR*, *NPPA*)

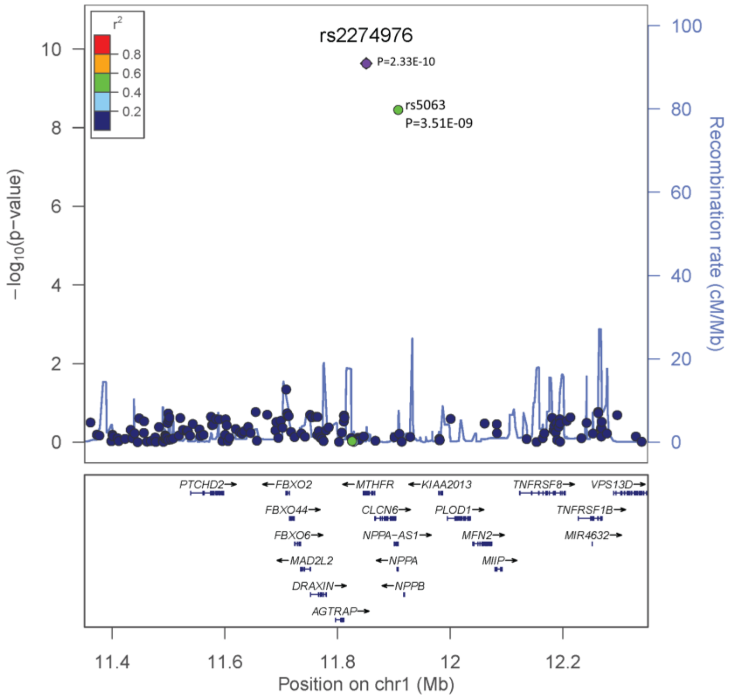

(b): 1q21 (*LCE3D-LCE3E*)

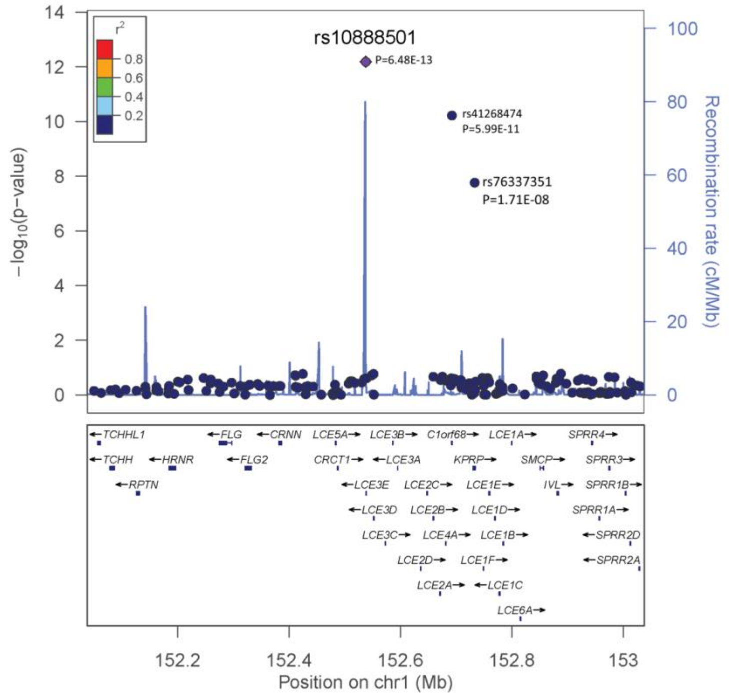

(c): 2q12.1(*IL1RL1*)

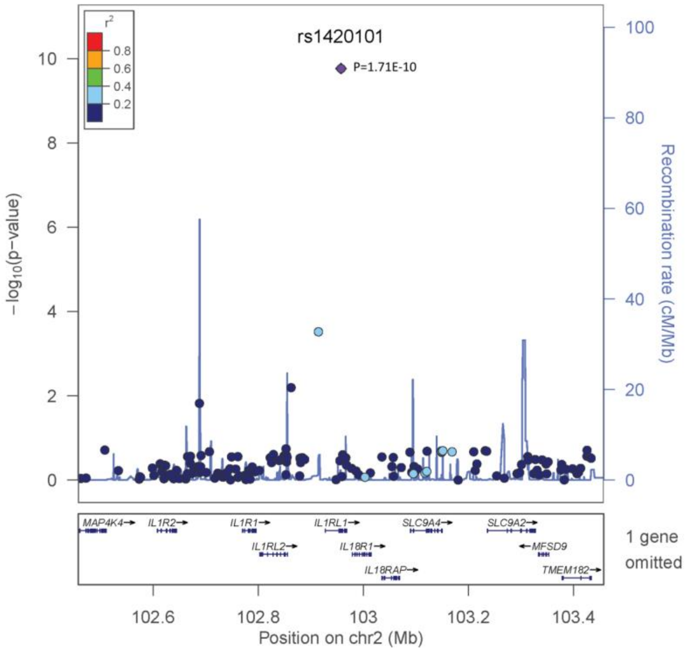

(d): 5q33.3(*RNF145*)

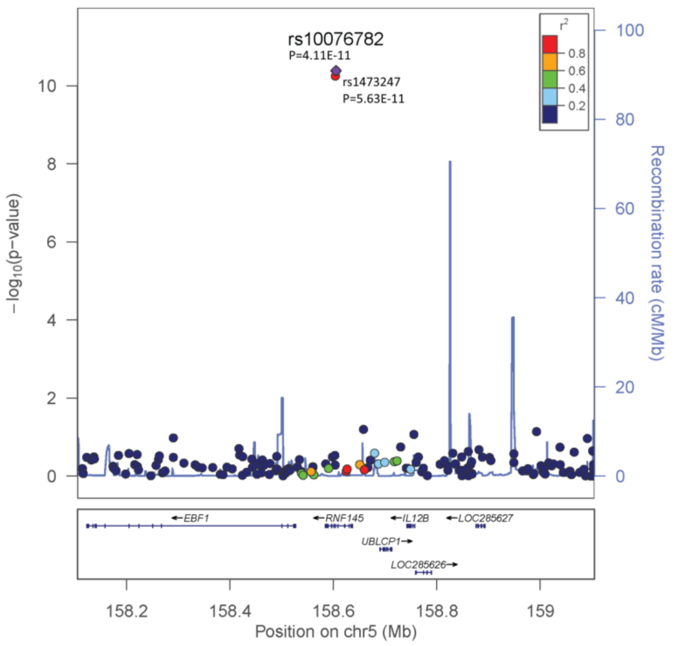

(e): 11p15.4 (*ZNF143*)

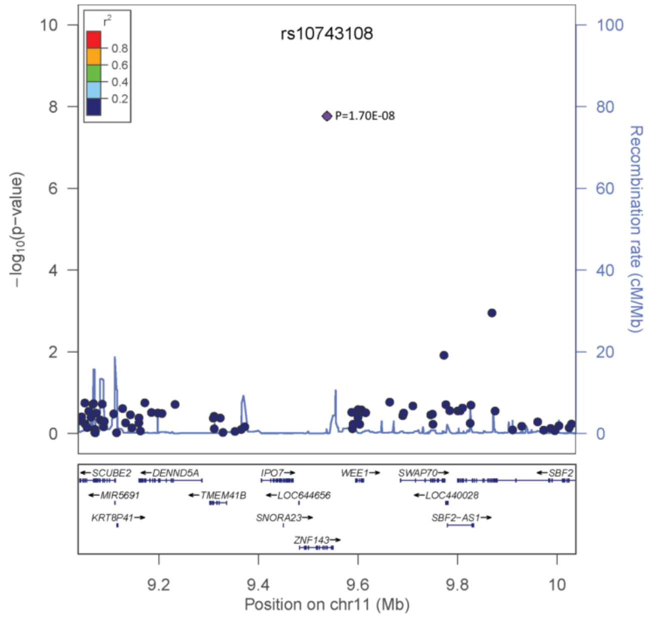

(f): 14q13 (*NFKBIA*)

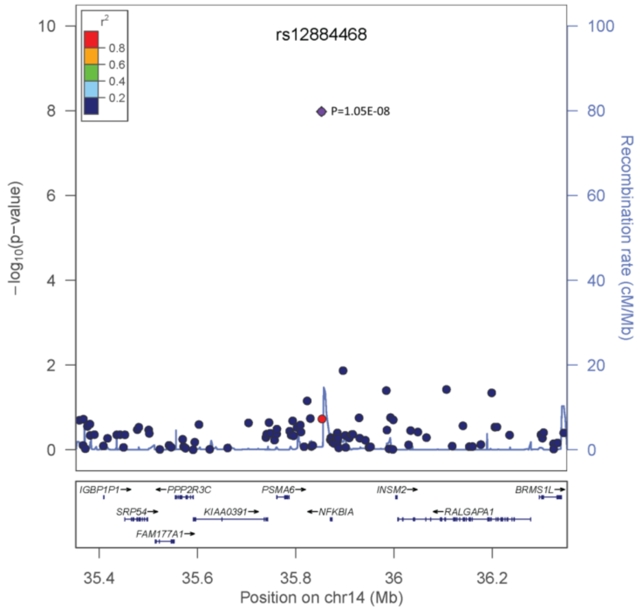

(g): 1p36.11 (*ZNF683*)

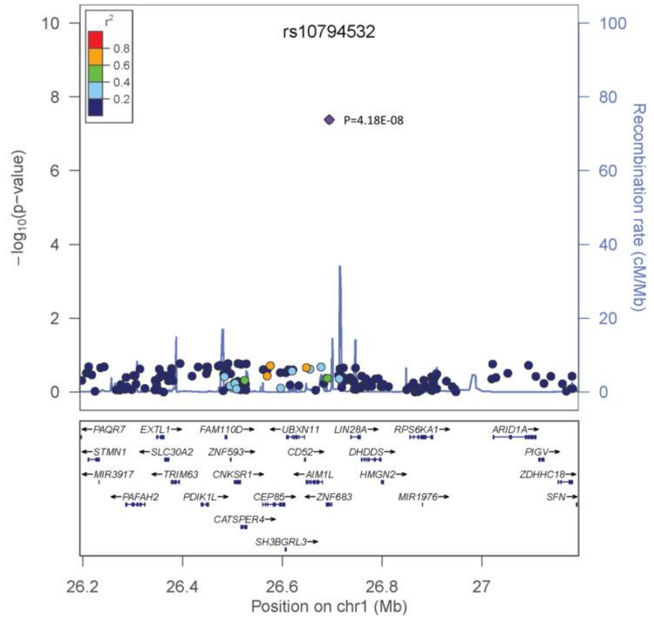

(h): 17q25.3 (*TMC6*)

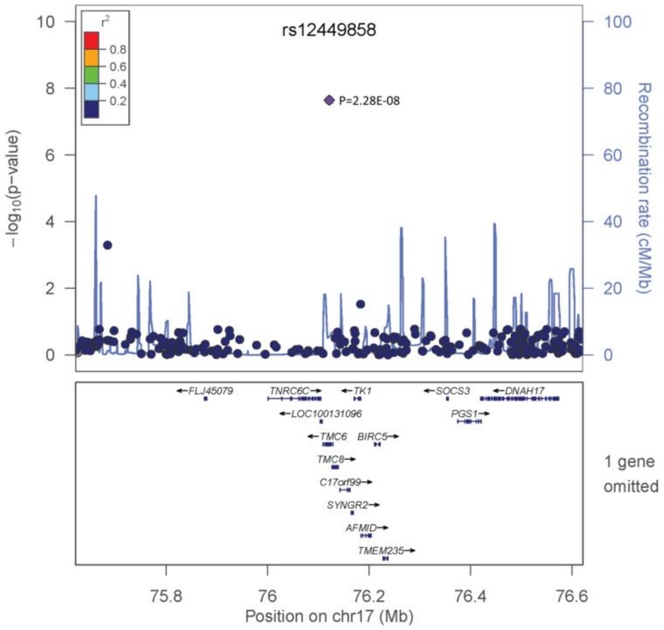

(i): 1p31.3 (*C1orf141*)

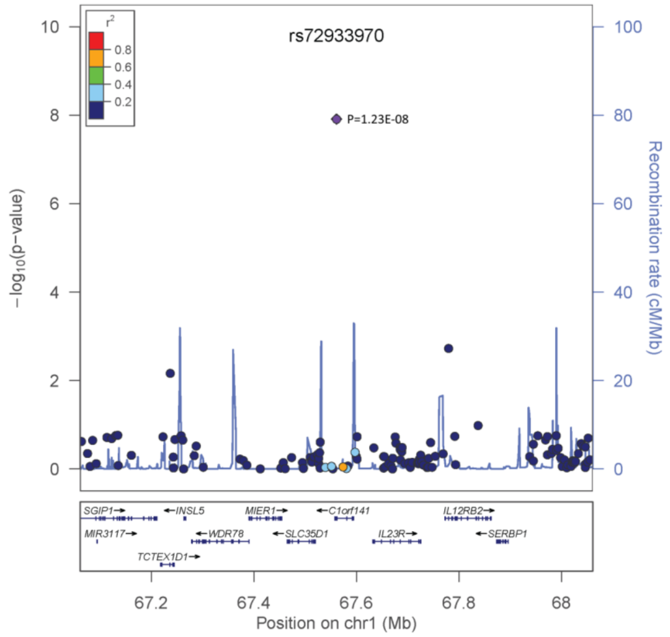

(j): 1q22 (*AIM2*)

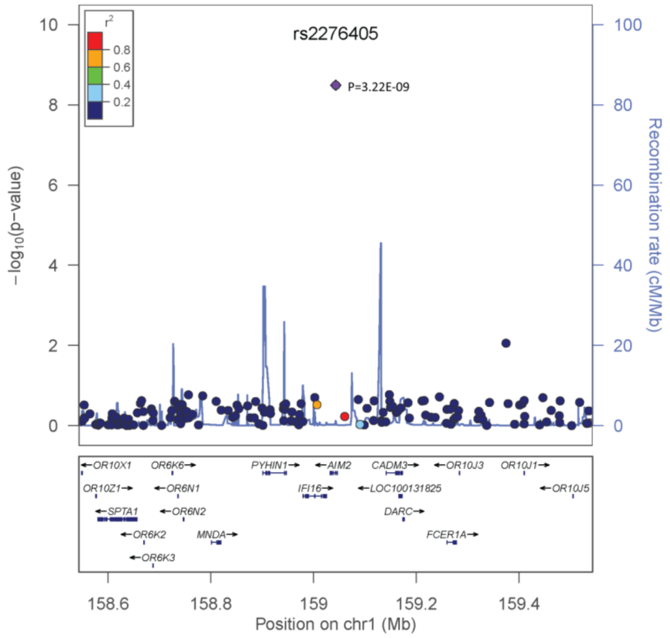

(k): 3q13 (*CASR*)

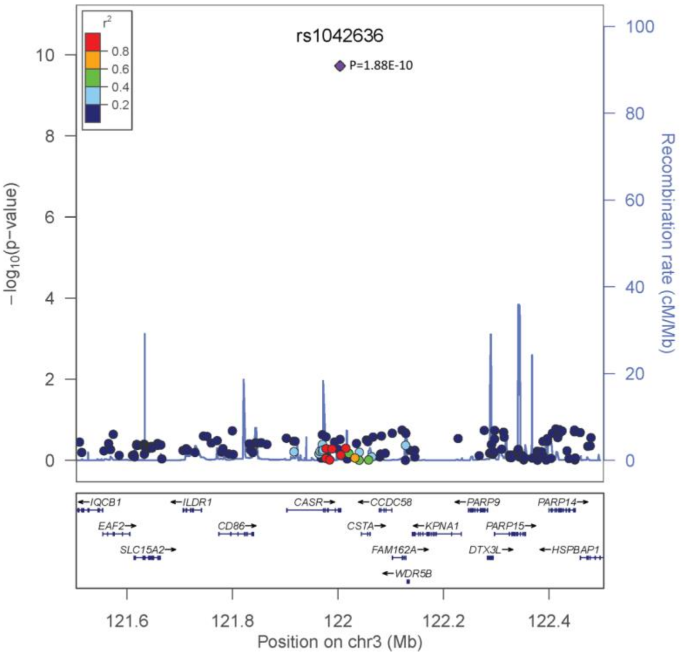

(l): 3q26.2-q27 (*GPR160*)

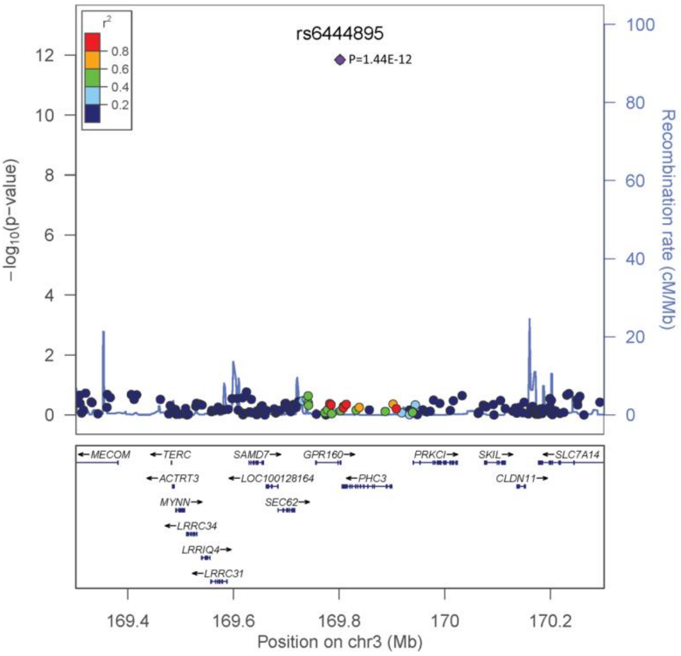

(m): 5q14 (*ZFYVE16*)

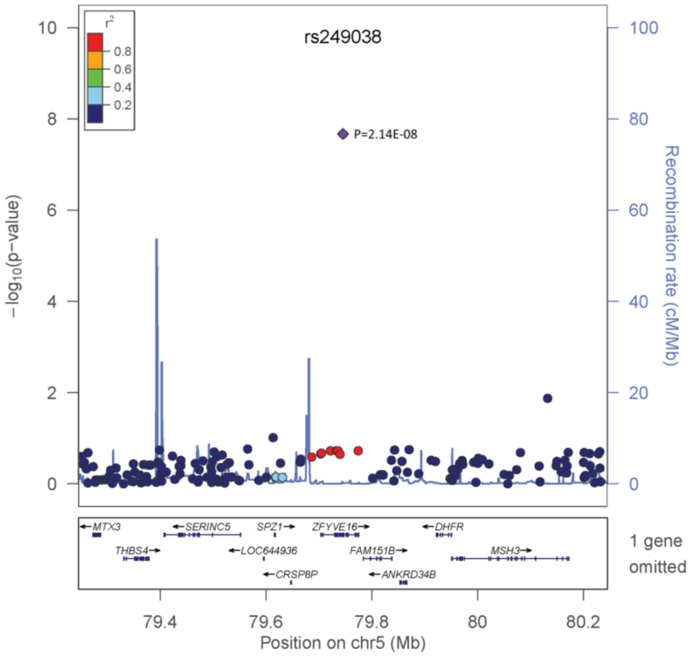

(n): 5q32-q33.1 (*TNIP1*)

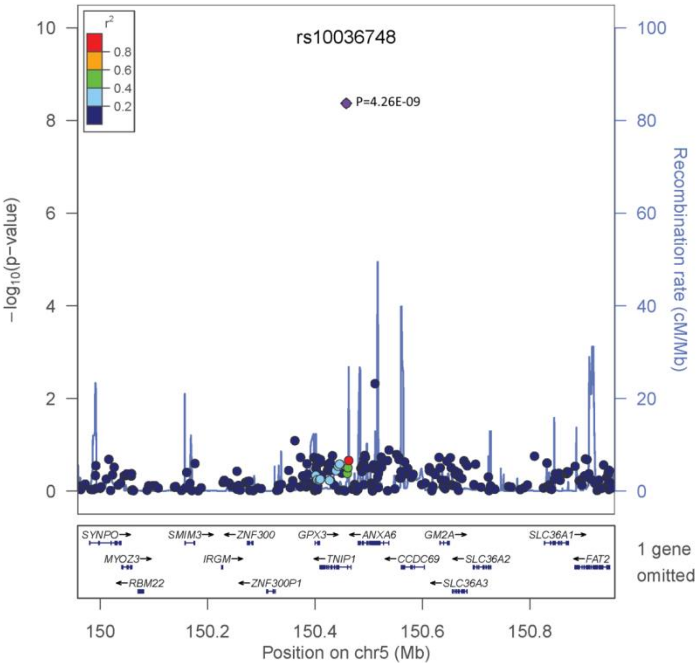

(o): 7p14.3 (*CCDC129*)

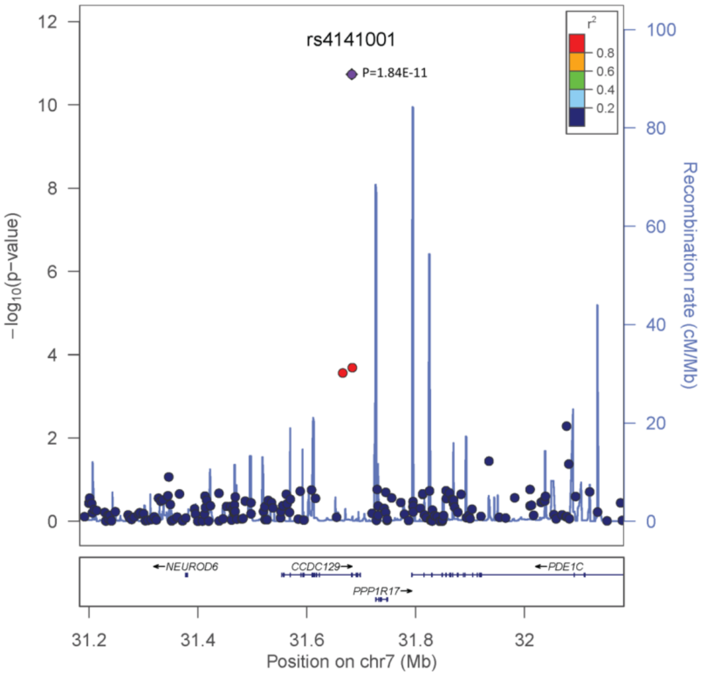

(p): 11q13.1 (*AP5B1*)

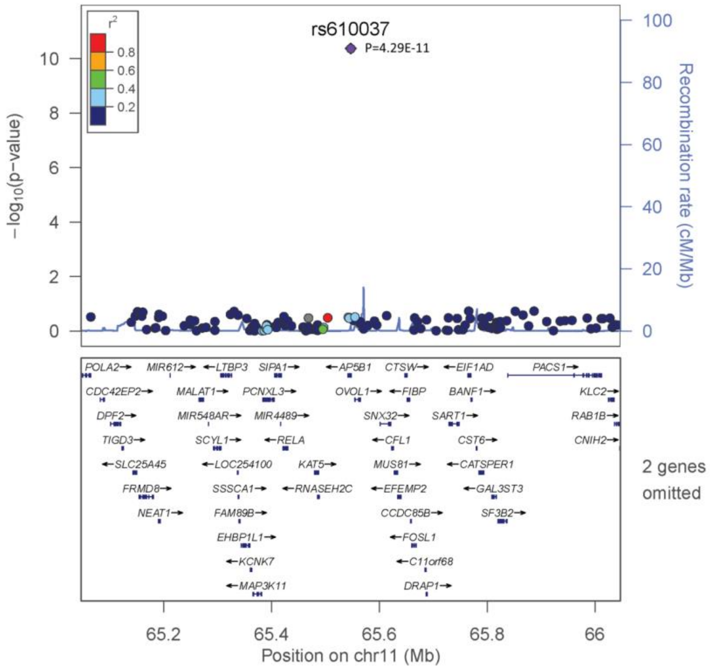

(q): 14q23.2(*SYNE2*)

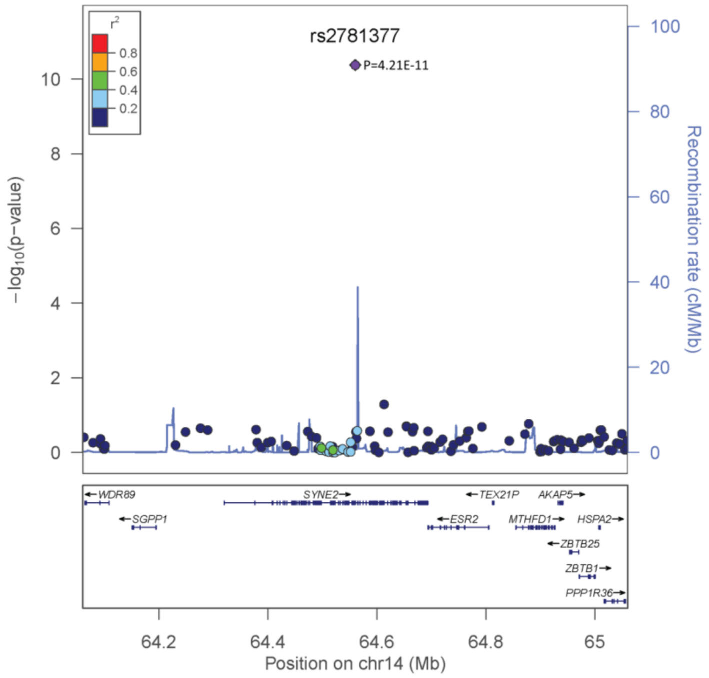

(r): 21q22.11 (*IFNGR2*)

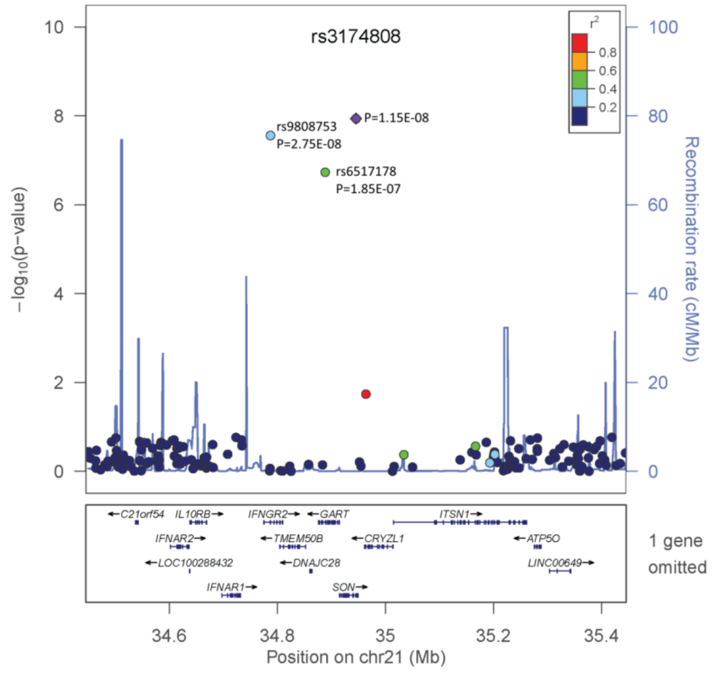

(s): 1q42.3(*TARBP1*)

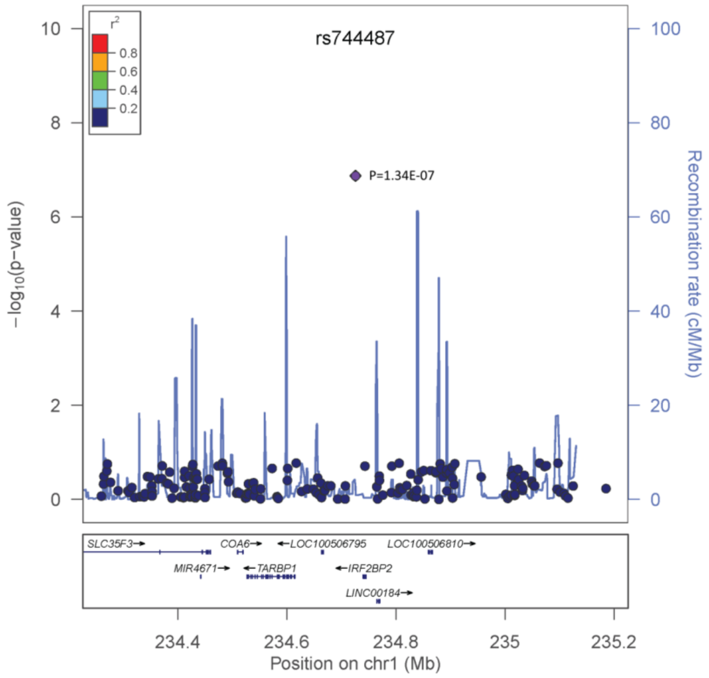

(t): 10q22.3 (*ZMIZ1*)

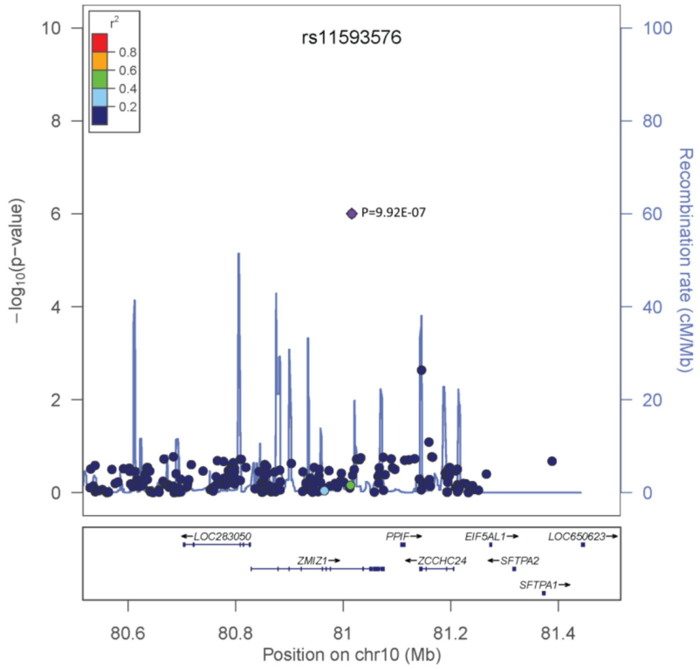

Supplementary Figure 5 | The scatter plots of the association evidences within 13 loci for psoriasis identified in the current study. The P values of SNPs (shown as –

log10 values in y-axis, from single marker based association analysis using the logistic regression) were plotted against their map positions (x-axis). The color of each SNP spot reflects its r^2 with the top SNP (large blue diamond) within each association locus, changing from red to white. Estimated recombination rates (based on the targeted sequencing data) were plotted in light blue. Gene annotations were adapted from the University of California at Santa Cruz Genome Browser (http://genome.ucsc.edu/). (a): 1p36.3 (*MTHFR*, *NPPA*), (b):1q21 (*LCE3D-LCE3E*), (c): 2q12.1 (*IL1RL1*), (d): 5q33.3 (*RNF145*), (e): 11p15.4 (*ZNF143*), (f): 14q13 (*NFKBIA*), (g): 1p36.11 (*ZNF683*), (h): 17q25.3 (*TMC6*), (i): 1p31.3 (*C1orf141*), (j): 1q22 (*AIM2*), (k): 3q13 (*CASR*), (l): 3q26.2-q27 (*GPR160*), (m): 5q14 (*ZFYVE16*), (n): 5q32-q33.1 (*TNIP1*), (o): 7p14.3 (*CCDC129*), (p): 11q13.1 (*AP5B1*), (q): 14q23.2 (*SYNE2*), (r): 21q22.11 (*IFNGR2*), (s): 1q42.3 (*TARBP1*).

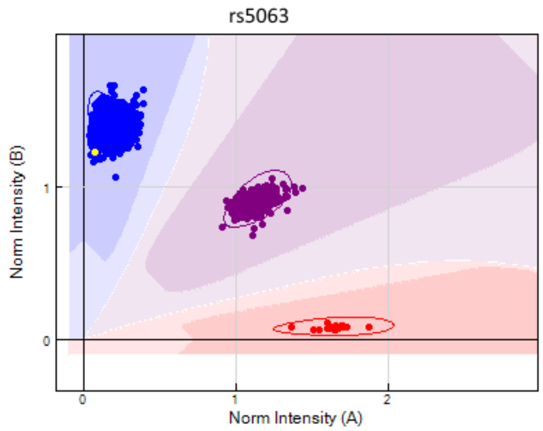
rs5063
Norm Intensity (B)
Norm Intensity (A)

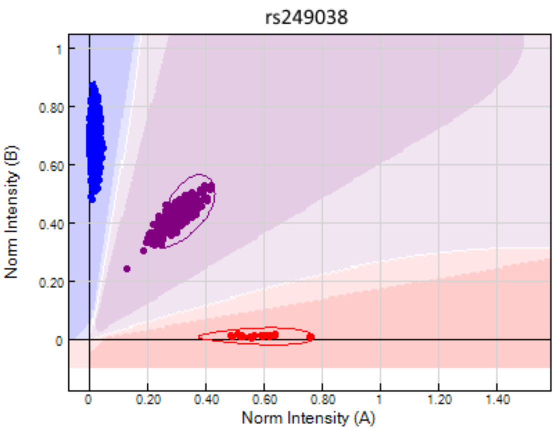
rs249038
Norm Intensity (B)
Norm Intensity (A)

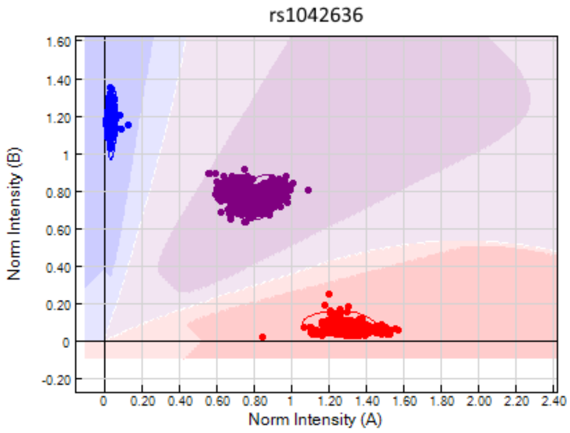
rs1042636
Norm Intensity (B)
Norm Intensity (A)

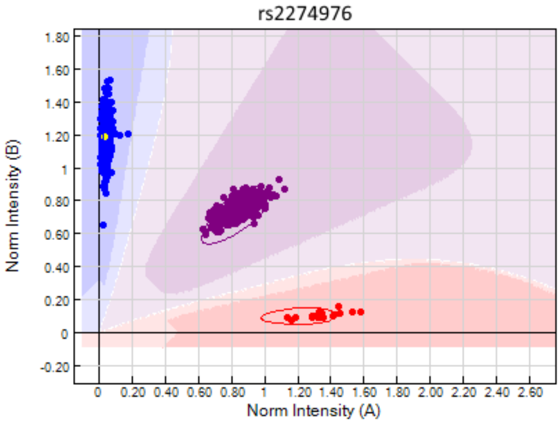
rs2274976
Norm Intensity (B)
Norm Intensity (A)

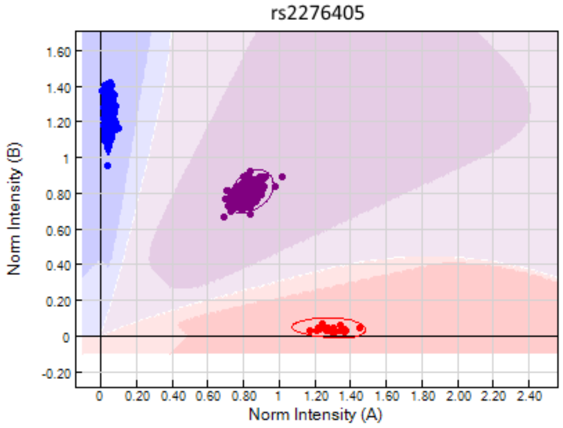
rs2276405
Norm Intensity (B)
Norm Intensity (A)

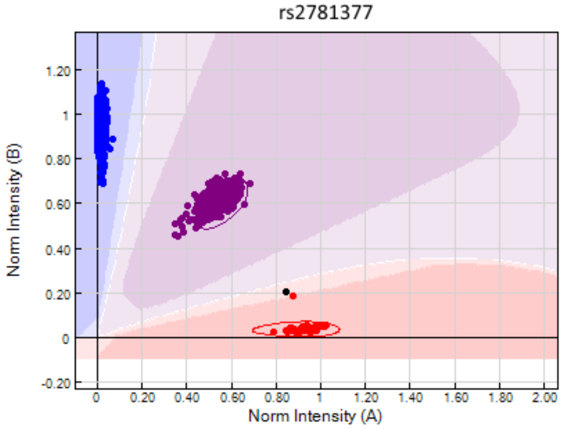
rs2781377
Norm Intensity (B)
Norm Intensity (A)

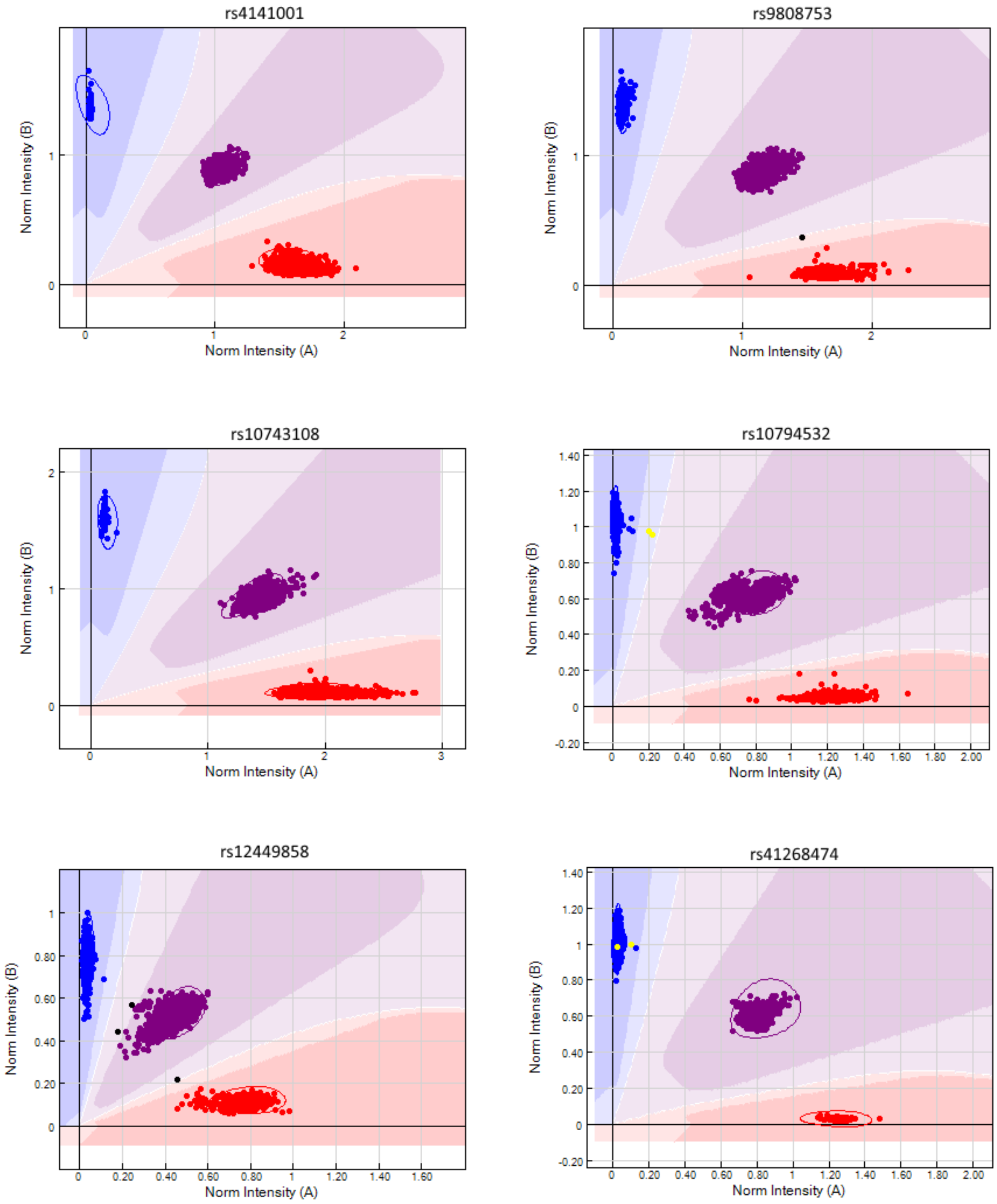
rs4141001
Norm Intensity (B)
Norm Intensity (A)
rs9808753
Norm Intensity (B)
Norm Intensity (A)
rs10743108
Norm Intensity (B)
Norm Intensity (A)
rs10794532
Norm Intensity (B)
Norm Intensity (A)
rs12449858
Norm Intensity (B)
Norm Intensity (A)
rs41268474
Norm Intensity (B)
Norm Intensity (A)

rs72933970

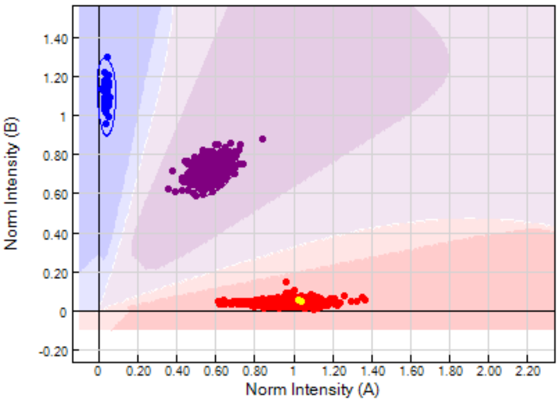

rs76337351

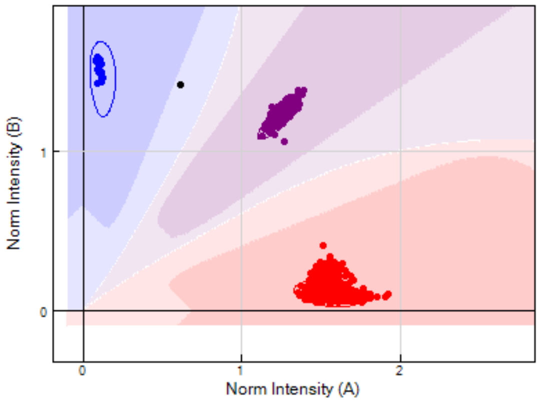

rs10076782

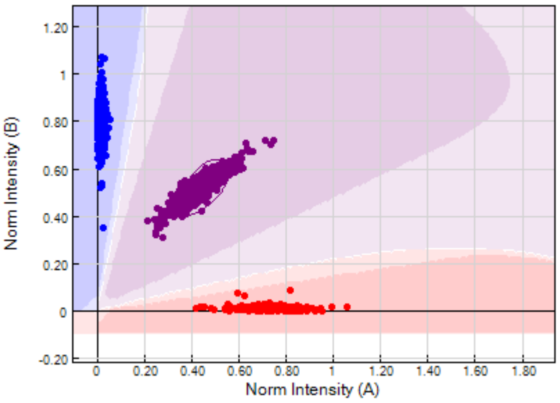

rs10888501

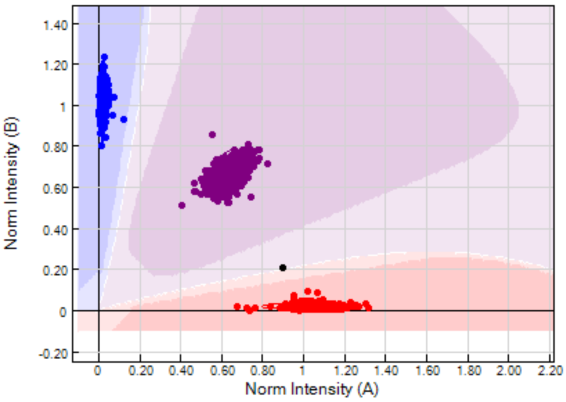

rs12884468

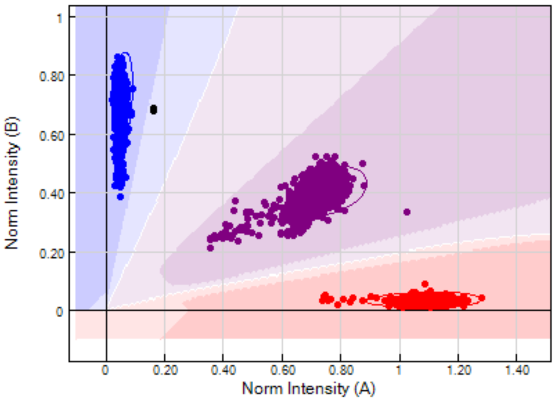

rs1420101

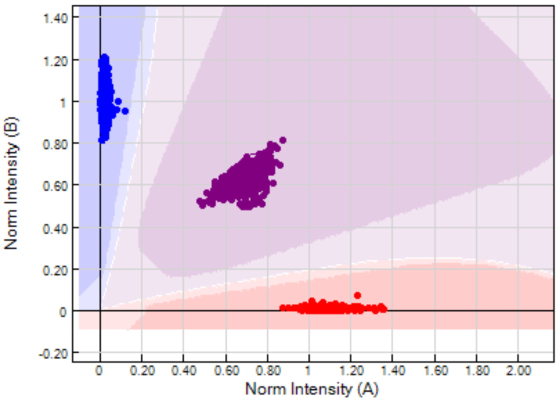

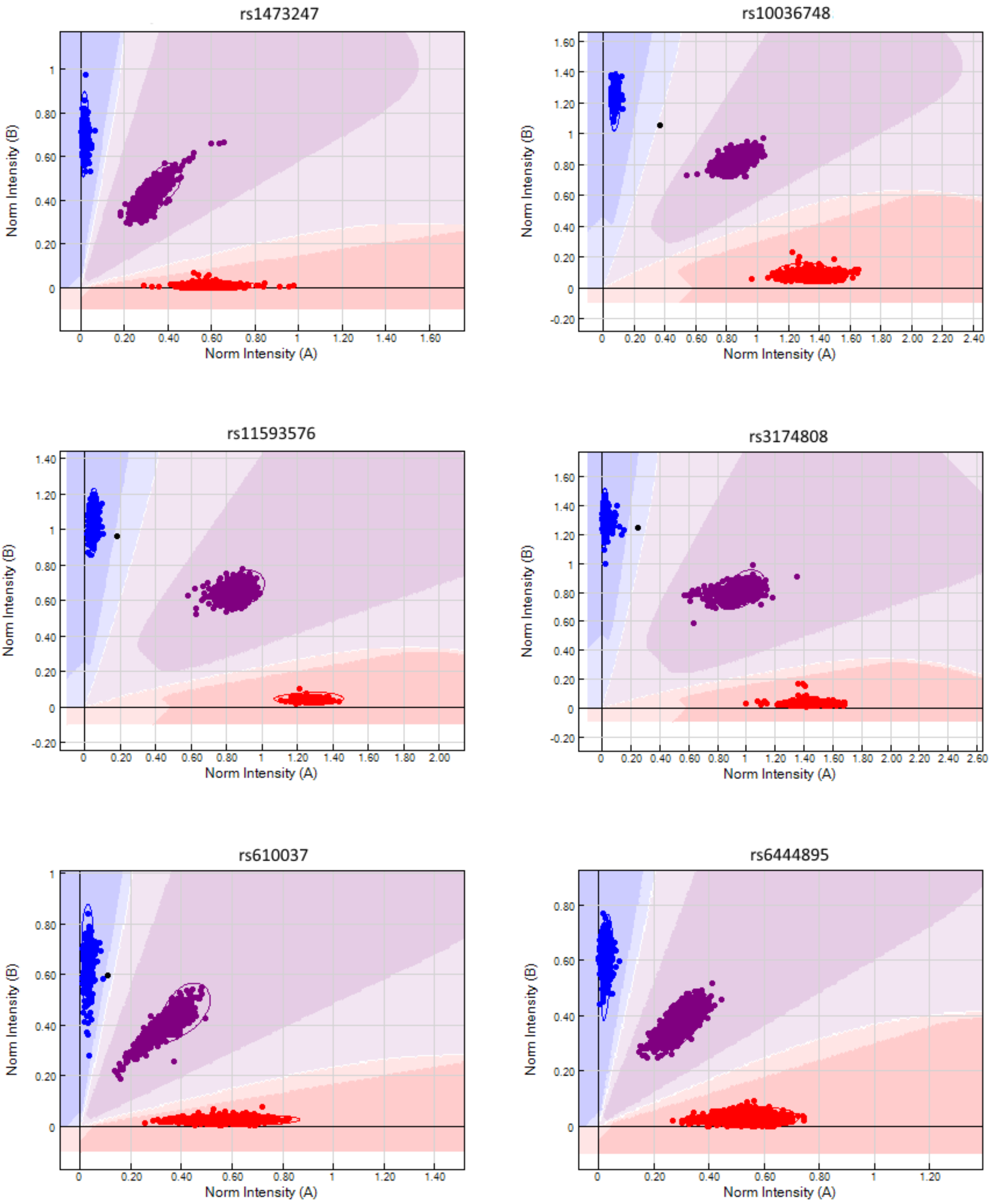
rs1473247
Norm Intensity (B)
Norm Intensity (A)
rs10036748
Norm Intensity (B)
Norm Intensity (A)
rs11593576
Norm Intensity (B)
Norm Intensity (A)
rs3174808
Norm Intensity (B)
Norm Intensity (A)
rs610037
Norm Intensity (B)
Norm Intensity (A)
rs6444895
Norm Intensity (B)
Norm Intensity (A)

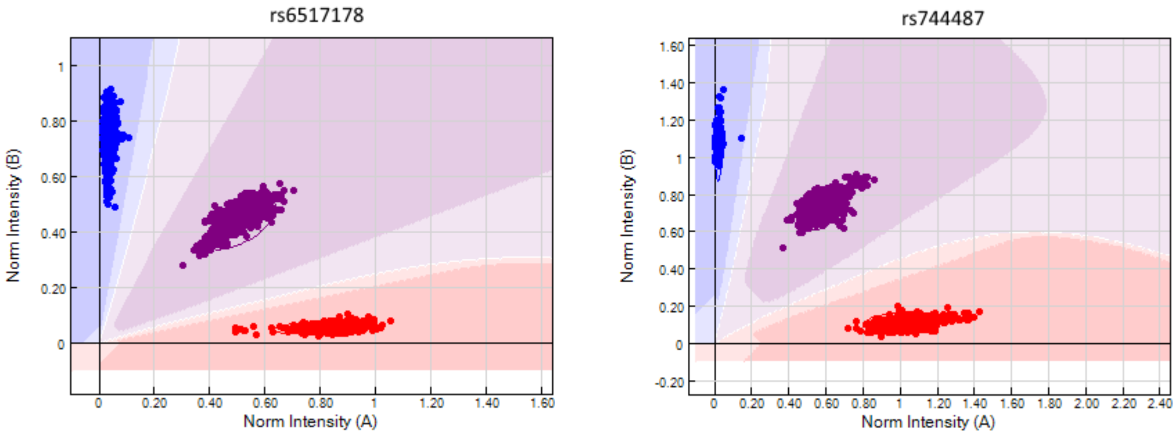

Supplementary Figure 6 | The genotype cluster plots for each SNP in association results from the first two stages, including 8,949 individuals (4,179 cases and 4,770 controls) in the first stage and 13,473 individuals (7,066 cases and 6,407 controls) in the second stage (Table 1), and three stages of combined analyses, including 8,949 individuals (4,179 cases and 4,770 controls) in the first stage, 13,473 individuals (7,066 cases and 6,407 controls) in the second stage and 20,338 individuals (6,369 cases and 13,969 controls) in the replication stage (Table 2).

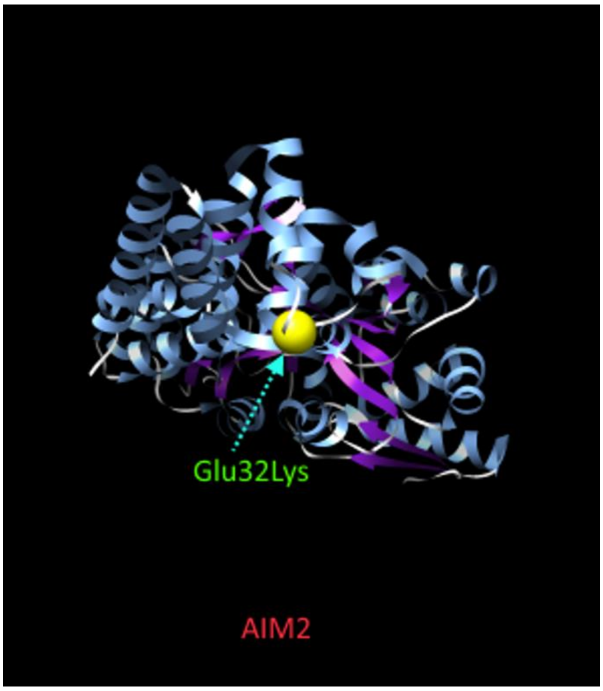

Supplementary Figure 7 | Crystal structures of the absent in melanoma 2 (*AIM2*). Amino acid residue 32 of *AIM2* (rs2276405) was found to be located in the middle of an alpha-helix motif buried inside the protein structure. The wild-type Glu residue is acidic, but the mutant Lys residue is basic. As the chemical properties of Glu and Lys are completely opposite, this substitution may destabilise the alpha-helix motif.

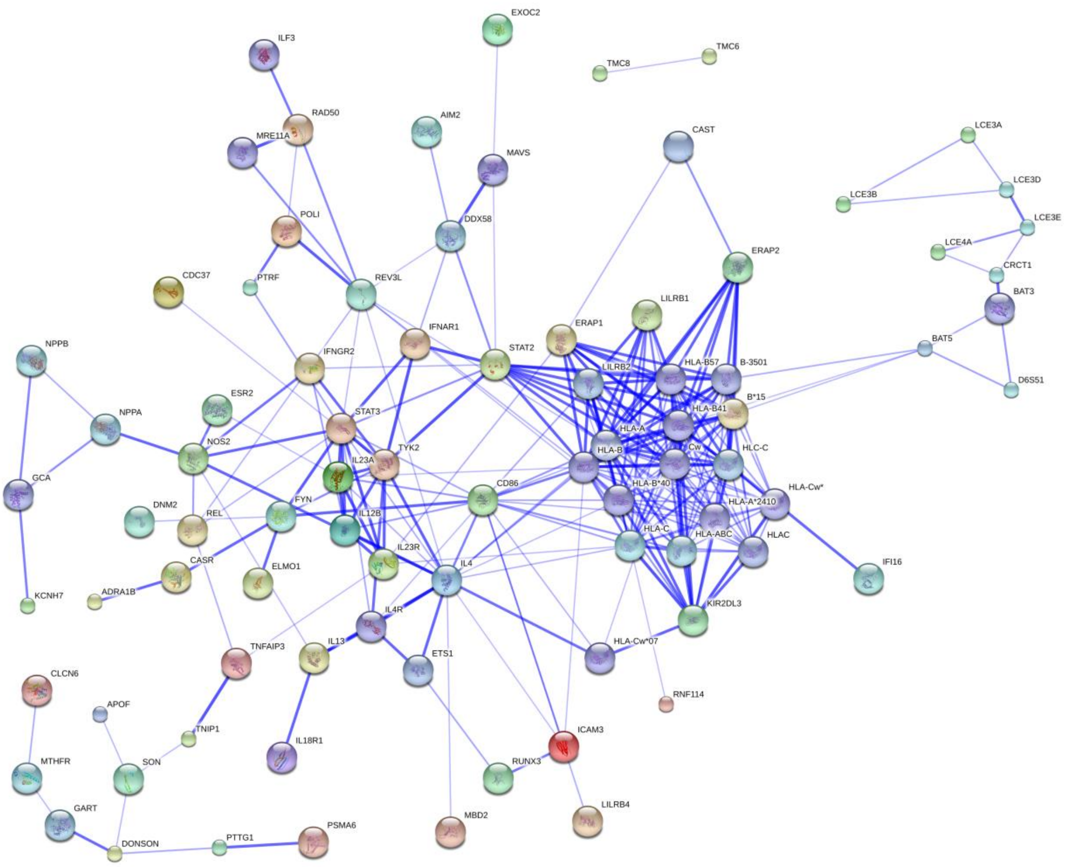

Supplementary Figure 8 | Functional similarity network of genes in psoriasis associated loci. The functional similarity network in this figure is constructed for the genes/loci reported in previous GWASs and present study. The network contains 5 out of the 15 non-HLA psoriasis genes from the present study, such as *MTHFR*, *NPPA*, *AIM2*, *CASR* and *IFNGR2*. Genes and their nodes that are not connected to any other node in the network are omitted.

Supplementary Tables:

Supplementary Table 1. Summary of the samples analyzed in this study.

	Exome_Asian Array		Exome_Fine Array		Genotyping	
	Cases	Controls	Cases	Controls	Cases	Controls
Sample size	4,179	4,770	7,066	6,407	6,369	13,969
Mean age(s.d.)	35.61±14.33	34.58±12.86	34.56±14.36	28.95±14.70	35.72±12.86	35.59±16.92
Mean age of onset(s.d.)	27.22±12.82	/	26.49±13.32	/	28.03±13.70	/
Male/Female	2,810/1,369	2,605/2,165	4,175/2,891	3,053/3,354	3,684/2,685	7,778/6,191

Supplementary Table 2. SNPs detected in Exome_Asian Array and Exome_Fine Array

Function	Exome_Asian Array*			Exome_Fine Array#		
	MAF <1%	MAF <5%	All	MAF <1%	MAF <5%	All
coding-notMod3	1,014	1,074	1,201	868	923	1,035
coding-notMod3-nearsplice	37	37	41	34	34	38
coding-synonymous	4,782	5,040	5,677	3,839	4,081	4,776
coding-synonymous-near-splice	2,211	2,289	2,427	1,997	2,087	2,223
intergenic	5,912	6,614	14,727	5,180	6,428	21,123
intron	6,682	7,261	12,777	5,626	6,701	20,898
missense	196,857	203,656	214,467	163,499	170,828	181,387
missense-near-splice	5,496	5,618	5,830	4,775	4,928	5,136
near-gene-3	39	57	284	36	71	443
near-gene-5	32	52	329	33	61	787
splice-3	730	742	766	594	608	633
splice-5	1,092	1,121	1,147	1,001	1,037	1,063
stop-gained	9,684	9,965	10,325	8,354	8,667	9,018
stop-gained-near-splice	275	283	294	240	249	260
stop-lost	211	217	234	140	142	156
utr-3	1,231	1,340	1,795	1,009	1,128	2,007
utr-5	689	731	886	573	614	797
Sum	236,974	246,097	273,207	197,798	208,587	251,780

***The number of monomorphism in Exome_Asian Array：274,959 - 273,207 = 1,752**

#The number of monomorphism in Exome_Fine Array：268,410 - 251,780 = 16,630

Supplementary Table 3. The association evidence at known GWAS loci in Exome_Asian Array and Exome_Fine Array stages through logistic regression (additive model), respectively.

CHR	SNP	BP	Function	Gene	Exome_Asian Array				*P*	OR	95% CI	Exome_Fine Array				*P*	OR	95% CI	Reported *P*_Value	Reported OR	Reported Ethnic	Reference PMID
					A1	F_A	F_U	A2				A1	F_A	F_U	A2							
1	Chr1:67,421,184	67648596	missense	*IL23R*	A	0.0397	0.0482	G	5.83E-03	0.82	0.71-0.94	A	0.0355	0.0482	G	2.11E-07	0.73	0.65-0.82	1.94E-11	0.72	Chinese	24212883
2	rs10865331	62551472	intergenic	*B3GNT2*	A	0.4211	0.4037	G	1.80E-02	1.08	1.01-1.14	A	0.4218	0.4049	G	4.88E-03	1.07	1.02-1.13	4.70E-10	1.12	European	23143594
17	rs11652075	78178893	missense	*CARD14*	A	0.4837	0.4924	G	2.44E-01	0.97	0.91-1.02	A	0.4771	0.5056	G	2.85E-06	0.89	0.85-0.94	3.40E-08	1.11	European	23143594
5	rs12188300	158829527	intergenic	*IL12B*	A	0.0012	0.0003	T	2.89E-02	3.81	1.05-13.84	A	0.0014	0.0002	T	9.15E-04	6.05	1.80-20.37	3.20E-53	1.58	European	23143594
19	rs12459008	53454789	missense	*ZNF816*	A	0.3209	0.3459	T	4.06E-04	0.89	0.84-0.95	A	0.3201	0.3534	T	7.91E-09	0.86	0.82-0.91	2.25E-09	0.88	Chinese	24212883
14	rs12586317	35682172	intron	*NFKBIA, PSMA6*	G	0.2966	0.3117	A	2.77E-02	0.93	0.87-0.99	G	0.3042	0.3155	A	4.62E-02	0.95	0.90-1.00	2.00E-08	1.15	European	20953189
19	rs12720356	10469975	missense	*TYK2*	C	0.0005	0.0000	A	3.26E-02	NA	NA	C	0.0004	0.0002	A	5.69E-01	1.51	0.36-6.33	4.00E-11	1.4	European	20953190
6	rs13190932	111913070	intron	*TRAF3IP2*	A	0.0020	0.0006	G	8.85E-03	3.24	1.28-8.22	A	0.0016	0.0009	G	1.52E-01	1.66	0.82-3.36	8.56E-17	1.83	European	20953816
22	rs181359	21928641	intron	*YDJC*	A	0.4755	0.4620	G	7.21E-02	1.06	1.00-1.12	A	0.4739	0.4718	G	7.35E-01	1.01	0.96-1.06	6.30E-13	0.84	European	22482804
5	rs20541	131995964	missense	*IL13*	A	0.2897	0.3026	G	5.95E-02	0.94	0.88-1.00	A	0.2859	0.3063	G	2.52E-04	0.91	0.86-0.96	5.00E-15	1.27	European	19169254
1	rs2201841	67694202	intron	*IL23R*	A	0.2691	0.2745	G	4.14E-01	0.97	0.91-1.04	A	0.2765	0.2731	G	5.33E-01	1.02	0.96-1.07	1.75E-10	1.23	European	19262574
5	rs2303138	96350710	missense	*LNPEP*	A	0.4893	0.4725	G	2.47E-02	1.07	1.01-1.13	A	0.4871	0.4741	G	3.35E-02	1.05	1.00-1.11	1.83E-13	1.16	Chinese	23897274
6	rs240993	96139250	intron	*TRAF3IP2*	G	0.4466	0.4625	A	3.39E-02	0.94	0.88-1.00	G	0.4411	0.4535	A	4.15E-02	0.95	0.91-1.00	5.00E-20	1.25	European	20953190
5	rs26653	96118852	missense	*ERAP1*	G	0.4177	0.4349	C	2.05E-02	0.93	0.88-0.99	G	0.4230	0.4450	C	2.71E-04	0.91	0.87-0.96	5.27E-12	0.87	Chinese	24212883
5	rs27044	96101944	missense	*ERAP1*	C	0.4737	0.4918	G	1.53E-02	0.93	0.88-0.99	C	0.4754	0.4969	G	4.39E-04	0.92	0.87-0.96	2.16E-14	0.86	Chinese	24212883
5	rs27524	111913262	intron	*ERAP1*	A	0.4560	0.4423	G	6.54E-02	1.06	1.00-1.12	A	0.4543	0.4385	G	9.15E-03	1.07	1.02-1.12	3.00E-11	1.13	European	20953190
6	rs33980500	10463118	missense	*TRAF3IP2*	A	0.0034	0.0018	G	3.66E-02	1.88	1.03-3.44	A	0.0033	0.0018	G	1.37E-02	1.86	1.13-3.06	4.20E-45	1.52	European	23143594
19	rs34536443	111696091	missense	*TYK2*	G	0.0004	0.0004	C	8.39E-01	0.86	0.19-3.83	G	0.0001	0.0005	C	6.94E-02	0.26	0.05-1.25	9.10E-31	1.88	European	23143594

6	rs458017	64097233	missense	*TRAF3IP2*	G	0.0024	0.0009	A	1.62E-02	2.54	1.16-5.58	G	0.0025	0.0010	A	4.48E-03	2.45	1.29-4.62	2.00E-16	1.37	European	20953190
11	rs694739	61081542	intron	*PRDX5*	G	0.1451	0.1537	A	1.10E-01	0.94	0.86-1.02	G	0.1456	0.1544	A	4.31E-02	0.93	0.87-1.00	2.41E-14	0.89	European	22482804
2	rs702873	20763612	intron	*REL*	A	0.1344	0.1434	G	8.15E-02	0.93	0.85-1.01	A	0.1318	0.1376	G	1.67E-01	0.95	0.89-1.02	4.00E-09	1.12	European	20953190
13	rs72474224	35832666	missense	*GJB2*	A	0.0583	0.0510	G	3.13E-02	1.15	1.01-1.31	A	0.0576	0.0536	G	1.53E-01	1.08	0.97-1.20	7.46E-11	1.34	Chinese	24212883
14	rs8016947	31431780	intergenic	*NFKBIA*	A	0.4575	0.4828	C	7.18E-04	0.90	0.85-0.96	A	0.4588	0.4760	C	4.77E-03	0.93	0.89-0.98	2.00E-11	1.19	European	20953190
6	rs2395029	31431780	ncRNA	*HCP5*	C	0.0719	0.0274	A	9.12E-44	2.75	2.37-3.19	NA	NA	NA	NA	NA	NA	NA	2.00E-26	4.1	European	18369459

Supplementary Table 4. Conditional analysis the novel loci and reported loci in exome array and replication samples. *P* value were generated using logistic regression.

Chr	Gene	Variants IDs (hg18)	Allele	Exome array and replication samples					Logistic condition			LD	
				MAF		*P* value	OR	95% CI	Condition	*P* value	OR	D'	r^2
				Cases	Controls								
1p31.3	*C1orf141*	rs72933970	G/A	0.09	0.08	1.23E-08[a]	1.16	1.09-1.21	rs2201841	1.52E-04	1.14	0.13	0.004
									rs11209026	1.04E-03	1.12	0.00	0.0001
				0.089	0.077	2.98E-03[c]	1.18	1.06-1.32	chr1_67421184	7.93E-03	1.16	0.70	0.002
									rs3762318	4.22E-02	1.12	0.72	0.002
	IL23R	**rs2201841**[1, 2]	A/G	0.27	0.27	6.78E-01[b]	1.00	0.95-1.03	rs72933970	5.48E-01	0.99	0.13	0.004
									rs11209026	6.92E-01	0.99	0.10	0.0001
				0.28	0.27	4.1E-01[c]	1.02	0.96-1.10	chr1_67421184	.8.21E-01	1.01	0.78	0.01
									rs3762318	4.31E-01	0.93	0.81	0.011
		**rs11209026**[3, 4]	A/G	0.0001	0.0005	1.61E-01[b]	0.34	0.07-1.66	rs72933970	1.95E-01	0.35	0.00	0.0001
									rs2201841	1.94E-01	0.35	0.10	0.0001
				0.0001	0.0006	9.6E-02[c]	0.20	0.02-1.65	chr1_67421184	1.31E-01	0.20	0.06	0.0003
									rs3762318	3.91E-01	0.37	1	0.0001
		**chr1_67421184**[5]	A/G						rs72933970	1.13E-04	0.74	0.70	0.002
				0.037	0.049	8.56E-05[c]	0.74	0.63-0.86	rs3762318	2.31E-03	0.70	1.00	0.005
									rs2201841	8.50E-05	0.73	0.78	0.01
									rs11209026	7.63E-05	0.73	0.06	0.0003
		**rs3762318**[5]	A/G	0.040	0.048	5.82E-03[a]	0.82	0.70-0.94	rs72933970	1.29E-02	0.83	0.72	0.002
									chr1_67421184	2.81E-02	1.18	1.00	0.005
				0.036	0.046	5.71E-02[c]	0.77	0.59-1.01	rs2201841	5.40E-03	0.81	0.81	0.011
									rs11209026	5.65E-03	0.82	1	0.0001

1p36	*NPPA*	rs5063	A/G	0.06	0.07	3.51E-09 [a]	0.85	0.79-0.88	rs2274976	2.83E-01	0.94	0.70	0.49
	MTHFR	rs2274976	A/G	0.06	0.08	2.33E-10 [b]	0.79	0.71-0.83	rs5063	2.60E-05	0.80	0.70	0.49
1q21.3	*C1orf68*	rs41268474	A/G	0.10	0.09	5.987E-11 [a]	1.17	1.12-1.23	rs10888501	5.20E-08	1.15	0.48	0.02
									rs76337351	4.42E-05	1.15	0.10	0.01
									rs4085613	3.03E-02	1.08	0.89	0.05
									rs4112788	3.25E-02	1.08	0.89	0.05
	KPRP	rs76337351	G/C	0.05	0.06	1.71E-08 [a]	0.83	0.77-0.88	rs10888501	2.36E-07	0.85	0.07	0.0003
									rs41268474	4.91E-05	0.84	0.10	0.01
									rs4085613	5.07E-01	0.97	0.93	0.07
									rs4112788	5.54E-01	0.97	0.92	0.07
		rs10888501	G/A	0.46	0.50	1.382E-13 [b]	0.87	0.83-0.90	rs41268474	1.50E-11	0.87	0.48	0.02
									rs76337351	1.63E-13	0.86	0.07	0.0003
									rs4085613	3.69E-01	1.02	0.79	0.45
									rs4112788	3.12E-01	1.03	0.79	0.45
	LCE3D-LCE3E	**rs4085613**[6]	A/C	0.36	0.42	1.05E-34 [b]	0.78	0.75-0.81	rs10888501	1.12E-22	0.76	0.79	0.44
									rs41268474	1.11E-30	0.79	0.89	0.05
									rs76337351	1.10E-32	0.78	0.93	0.07
									rs4112788	8.44E-01	1.03	0.99	0.97
		**rs4112788**[7]	A/C	0.36	0.42	6.72E-36 [b]	0.78	0.75-0.81	rs10888501	8.23E-24	0.76	0.79	0.44
									rs41268474	8.26E-32	0.78	0.89	0.05
									rs76337351	6.67E-34	0.77	0.92	0.07
									rs4085613	2.38E-02	0.75	0.99	0.97
5q32-q33.1	*TNIP1*	rs10036748	G/A	0.25	0.24	4.26E-09 [a]	1.10	1.06-1.13	rs17728338	2.04E-03	1.07	0.23	0.02
		**rs17728338**[1, 2]	A/G	0.12	0.10	5.15E-17 [b]	1.30	1.22-1.38	rs10036748	2.79E-16	1.29		
5q33.3	*RNF145*	rs1473247	G/A	0.32	0.35	5.628E-11 [b]	0.88	0.84-0.91	rs10076782	NA	NA	0.95	0.91
									rs2082412	2.18E-02	0.94	0.69	0.34

									rs2546890	9.70E-05	0.92	0.52	0.13
									rs3213094	1.89E-02	0.94	0.60	0.26
		rs10076782	A/G	0.32	0.35	4.11E-11 [b]	0.88	0.84-0.91	rs1473247	NA	NA	0.95	0.91
									rs2082412	1.69E-02	0.94	0.68	0.34
									rs2546890	6.42E-05	0.92	0.51	0.12
									rs3213094	1.48E-02	0.94	0.60	0.26
		**rs2082412**[8]	A/G	0.39	0.43	1.72E-15 [b]	0.85	0.82-0.89	rs1473247	5.20E-07	0.88	0.69	0.34
									rs10076782	6.09E-07	0.88	0.68	0.34
									rs2546890	4.76E-05	0.91	0.68	0.30
									rs3213094	9.10E-01	1.01	0.91	0.82
	IL12B	**rs2546890**[9]	A/G	0.50	0.46	7.59E-16 [b]	1.17	1.13-1.22	rs1473247	5.51E-10	1.14	0.52	0.13
									rs10076782	6.10E-10	1.14	0.51	0.12
									rs2082412	4.10E-06	1.11	0.68	0.30
									rs3213094	3.66E-04	1.09	0.77	0.39
		**rs3213094**[10]	A/G	0.39	0.43	7.52E-19 [b]	0.84	0.81-0.87	rs1473247	4.07E-10	0.86	0.60	0.26
									rs10076782	4.74E-10	0.87	0.60	0.26
									rs2082412	1.52E-04	0.84	0.91	0.82
									rs2546890	3.99E-06	0.89	0.77	0.39
14q13	*NFKBIA*	rs12884468	A/G	0.46	0.48	1.84E-11 [b]	0.88	0.87-0.94	rs8016947	2.99E-10	0.89	0.62	0.35
									rs12586317	6.43E-06	0.91	0.64	0.21
		**rs8016947**[11]	A/C	0.44	0.47	1.53E-05 [b]	0.92	0.89-0.94	rs12884468	3.34E-01	0.98	0.62	0.35
									rs12586317	2.40E-03	0.94	0.55	0.16
	KIAA0391	**rs12586317**[12]	G/A	0.30	0.31	3.69E-03 [b]	0.94	0.90-0.98	rs8016947	3.66E-01	0.98	0.64	0.21
									rs12884468	5.70E-01	0.987	0.163	0.552
21q22.11	*IFNGR2*	rs9808753	G/A	0.42	0.44	2.75E-08 [b]	0.92	0.87-0.94	rs3174808	1.22E-04	0.94	0.76	0.15
	SON	rs3174808	G/A	0.26	0.25	1.15E-08 [b]	1.10	1.03-1.13	rs9808753	9.03E-04	1.06		

MAF, minor allele frequency

Allele, minor allele/major allele

a, including the Exome array stage and replication stage samples

b, only including the Exome array stage samples

c, 8,555 samples including 3,903 cases and 4,652 controls overlap the target sequence and the Exome array stage

Supplementary Table 5. Association results of 11 of 26 newly identified SNPs in previous GWAS study

CHR	SNP	BP	A1	F_A	F_U	A2	*P*_GWAS	OR	L95	U95	*P*_hwe
5	rs10076782	158604963	A	0.3292	0.3601	G	0.02	0.87	0.78	0.98	0.3988
3	rs1042636	122003769	G	0.4860	0.4935	A	0.58	0.97	0.87	1.08	0.4664
1	rs10794532	26694260	A	0.4930	0.4616	G	0.02	1.13	1.02	1.26	0.241
10	rs11593576	81015896	A	0.2078	0.1965	G	0.30	1.07	0.94	1.22	0.4419
2	rs1420101	102957716	A	0.3371	0.3563	G	0.14	0.92	0.82	1.03	0.6719
1	rs2274976	11850927	A	0.0663	0.0712	G	0.48	0.93	0.75	1.14	0.8535
5	rs249038	79745469	A	0.0505	0.0638	G	0.04	0.78	0.62	0.99	0.217
7	rs4141001	31682453	G	0.0918	0.1086	A	0.04	0.83	0.69	0.99	0.8015
1	rs5063	11907648	A	0.0645	0.0711	G	0.33	0.90	0.73	1.11	0.3561
21	rs6517178	34888621	A	0.3960	0.3622	G	0.01	1.16	1.04	1.29	0.5993
21	rs9808753	34787312	G	0.4175	0.4489	A	0.02	0.88	0.79	0.98	0.1161

Supplementary Table 6. The predictive value by sorting intolerant from tolerant (SIFT) and polymorphism phenotyping (PolyPhen) software.

Chr	Genes of interest	Variants ID	Function	Allele	F_A	F_U	SIFT Prediction	SIFT Score	PolyPhen2 Score
1p31.3	*C1orf141*	rs72933970	missense	G/A	0.0927	0.0781	TOLERATED	0.13	**N/A**
1p36.11	*ZNF683*	rs10794532	missense	A/G	0.4798	0.4562	DAMAGING	0	**N/A**
1p36.21	*NPPA*	rs5063	missense	A/G	0.0605	0.0751	TOLERATED	0.13	**N/A**
1p36.3	*MTHFR*	rs2274976	missense	A/G	0.0609	0.0769	DAMAGING	0	**N/A**
1q21.3	*C1orf68*	rs41268474	missense	A/G	0.0967	0.0866	TOLERATED	1	**0.002**
1q21.3	*KPRP*	rs76337351	missense	G/C	0.0498	0.0584	TOLERATED	0.06	**0.999**
1q22	*AIM2*	rs2276405	stop-gained	A/G	0.0546	0.0649	DAMAGING	0	**N/A**
3q13	*CASR*	rs1042636	missense	G/A	0.4672	0.4952	DAMAGING	0.01	**0.319**
5q14	*ZFYVE16*	rs249038	missense	A/G	0.0518	0.0616	TOLERATED	1	**0**
7p14.3	*CCDC129*	rs4141001	missense	G/A	0.0942	0.1112	TOLERATED	0.5	**N/A**
11p15.4	*ZNF143*	rs10743108	missense	C/G	0.2411	0.2224	TOLERATED	0.83	**0**
14q23.2	*SYNE2*	rs2781377	stop-gained	A/G	0.0869	0.1002	DAMAGING	0	**N/A**
17q25.3	*TMC6*	rs12449858	missense	A/G	0.2968	0.2759	DAMAGING	0.02	**1**
21q22.11	*IFNGR2*	rs9808753	missense	G/A	0.4249	0.4459	TOLERATED	1	**0.001**
21q22.11	*SON*	rs3174808	missense	G/A	0.2646	0.2448	N/A	N/A	**N/A**

Supplementary Table 7. The different expression levels of newly identified and conformedgenes in skin from patients and controls based on public psoriasis databases.

ID	$P_{(NN\text{-}PN)adj}$	$P_{(NN\text{-}PN\text{-}PP)adj}$	$P_{(NN\text{-}PP)adj}$	$P_{(PN\text{-}PP)adj}$	Genesymbol
207196_s_at	5.89E-03	7.89E-23	2.95E-13	3.84E-18	*TNIP1*
201502_s_at	7.61E-02	1.42E-02	4.92E-03	7.49E-01	*NFKBIA*
207901_at	4.00E-01	4.07E-21	3.14E-14	3.72E-12	*IL12B*
224328_s_at	6.37E-04	1.57E-73	2.99E-69	1.98E-43	*LCE3D*
206513_at	5.79E-01	5.11E-37	3.45E-26	7.70E-21	*AIM2*
210577_at	9.19E-01	2.00E-03	9.62E-04	7.48E-03	*CASR*
201642_at	2.35E-03	1.32E-12	4.05E-05	1.13E-11	*IFNGR2*
239035_at	3.56E-01	1.97E-09	6.74E-10	8.11E-06	*MTHFR*
202761_s_at	9.46E-01	1.42E-31	5.93E-25	1.18E-22	*SYNE2*
204328_at	1.91E-01	6.26E-05	6.60E-06	1.62E-02	*TMC6*
207526_s_at	6.12E-01	4.02E-05	9.17E-04	4.00E-04	*IL1RL1*
223423_at	1.04E-02	3.63E-05	1.05E-05	3.64E-01	*GPR160*
231858_x_at	5.41E-01	1.88E-05	8.26E-06	1.20E-03	*AP5B1*
214988_s_at	8.63E-01	3.14E-26	4.43E-21	4.65E-18	*SON*
202813_at	3.77E-01	4.72E-01	4.59E-01	7.58E-01	*TARBP1*
212124_at	3.85E-01	3.29E-23	5.73E-19	6.84E-15	*ZMIZ1*
212379_at	6.22E-01	2.90E-42	1.57E-32	7.3E-28	*GART*
1554638_at	3.31E-01	6.56E-07	3.91E-08	1.73E-04	*ZFYVE16*
221873_at	1.69E-01	4.49E-04	2.28E-05	7.25E-02	*ZNF143*
230756_at	1.68E-03	5.34E-09	8.90E-10	5.87E-02	*ZNF683*
209957_s_at	1.07E-02	3.21E-03	6.15E-03	8.86E-01	*NPPA*
234027_at	2.86E-01	3.49E-01	6.29E-01	4.64E-01	*CCDC129*
